# A cross-package Bioconductor workflow for analysing methylation array data

**DOI:** 10.12688/f1000research.8839.3

**Published:** 2017-04-05

**Authors:** Jovana Maksimovic, Belinda Phipson, Alicia Oshlack

**Affiliations:** 1 Murdoch Childrens Research Institute, Royal Children’s Hospital, Melbourne, Australia; 2 School of BioSciences, University of Melbourne, Melbourne, Australia; 3 School of Physics, University of Melbourne, Melbourne, Australia

**Keywords:** methylation, bioconductor, workflow, array

## Abstract

Methylation in the human genome is known to be associated with development and disease. The Illumina Infinium methylation arrays are by far the most common way to interrogate methylation across the human genome. This paper provides a Bioconductor workflow using multiple packages for the analysis of methylation array data. Specifically, we demonstrate the steps involved in a typical differential methylation analysis pipeline including: quality control, filtering, normalization, data exploration and statistical testing for probe-wise differential methylation. We further outline other analyses such as differential methylation of regions, differential variability analysis, estimating cell type composition and gene ontology testing. Finally, we provide some examples of how to visualise methylation array data.

## Introduction

DNA methylation, the addition of a methyl group to a CG dinucleotide of the DNA, is the most extensively studied epigenetic mark due to its role in both development and disease ( Bird, 2002; Laird, 2003). Although DNA methylation can be measured in several ways, the epigenetics community has enthusiastically embraced the Illumina HumanMethylation450 (450k) array ( Bibikova *et al.*, 2011) as a cost-effective way to assay methylation across the human genome. More recently, Illumina has increased the genomic coverage of the platform to >850,000 sites with the release of their MethylationEPIC (850k) array. As methylation arrays are likely to remain popular for measuring methylation for the foreseeable future, it is necessary to provide robust workflows for methylation array analysis.

**Figure 1.**
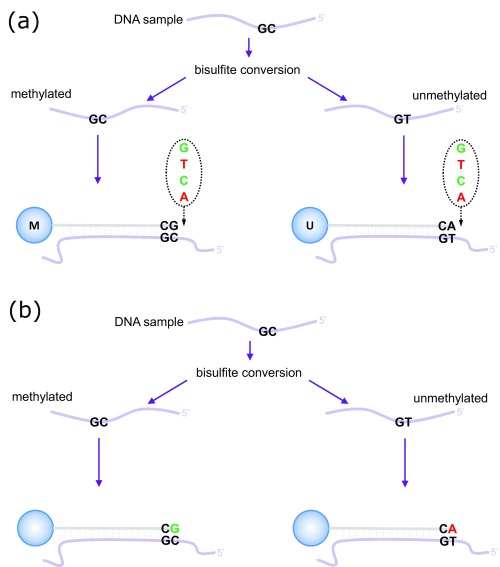
Illumina Infinium HumanMethylation450 assay, reproduced from Maksimovic *et al.*, 2012. ( **a**) Infinium I assay. Each individual CpG is interrogated using two bead types: methylated (M) and unmethylated (U). Both bead types will incorporate the same labeled nucleotide for the same target CpG, thereby producing the same color fluorescence. The nucleotide that is added is determined by the base downstream of the “C” of the target CpG. The proportion of methylation can be calculated by comparing the intensities from the two different probes in the same color. ( **b**) Infinium II assay. Each target CpG is interrogated using a single bead type. Methylation state is detected by single base extension at the position of the “C” of the target CpG, which always results in the addition of a labeled “G” or “A” nucleotide, complementary to either the “methylated” C or “unmethylated” T, respectively. Each locus is detected in two colors, and methylation status is determined by comparing the two colors from the one position.



Measurement of DNA methylation by Infinium technology (Infinium I) was first employed by Illumina on the HumanMethylation27 (27k) array ( Bibikova *et al.*, 2009), which measured methylation at approximately 27,000 CpGs, primarily in gene promoters. Like bisulfite sequencing, the Infinium assay detects methylation status at single base resolution. However, due to its relatively limited coverage the array platform was not truly considered “genome-wide” until the arrival of the 450k array. The 450k array increased the genomic coverage of the platform to over 450,000 gene-centric sites by combining the original Infinium I assay with the novel Infinium II probes. Both assay types employ 50bp probes that query a [C/T] polymorphism created by bisulfite conversion of unmethylated cytosines in the genome, however, the Infinium I and II assays differ in the number of beads required to detect methylation at a single locus. Infinium I uses two bead types per CpG, one for each of the methylated and unmethylated states ( Figure 1a). In contrast, the Infinium II design uses one bead type and the methylated state is determined at the single base extension step after hybridization ( Figure 1b). The 850k array also uses a combination of the Infinium I and II assays but achieves additional coverage by increasing the size of each array; a 450k slide contains 12 arrays whilst the 850k has only 8.

Regardless of the Illumina array version, for each CpG, there are two measurements: a methylated intensity (denoted by *M*) and an unmethylated intensity (denoted by *U*). These intensity values can be used to determine the proportion of methylation at each CpG locus. Methylation levels are commonly reported as either beta values ( *β* = *M/*( *M* + *U*)) or M-values ( *M value* = *log*2( *M/U*)). For practical purposes, a small offset, *α*, can be added to the denominator of the *β* value equation to avoid dividing by small values, which is the default behaviour of the getBeta function in *minfi*. The default value for *α* is 100. It may also be desirable to add a small offset to the numerator and denominator when calculating M-values to avoid dividing by zero in rare cases, however the default getM function in *minfi* does not do this. Beta values and M-values are related through a logit transformation. Beta values are generally preferable for describing the level of methylation at a locus or for graphical presentation because percentage methylation is easily interpretable. However, due to their distributional properties, M-values are more appropriate for statistical testing ( Du *et al.*, 2010).

In this workflow, we will provide examples of the steps involved in analysing methylation array data using R ( R Core Team, 2014) and Bioconductor ( Huber *et al.*, 2015), including: quality control, filtering, normalisation, data exploration and probe-wise differential methylation analysis. We will also cover other approaches such as differential methylation analysis of regions, differential variability analysis, gene ontology analysis and estimating cell type composition. Finally, we will provide some examples of useful ways to visualise methylation array data.

## Differential methylation analysis

### Obtaining the data

All of the data used in this workflow can be downloaded and extracted in R using the download.file and untar functions, as shown below. Alternatively, the data can be manually downloaded from: https://figshare.com/articles/methylAnalysisDataV3_tar_gz/4800970.



# the URL for the data download
url <- 
"https://ndownloader.figshare.com/files/7896205"
*# download the data*
if(!file.exists("methylAnalysisDataV3.tar.gz")){
	download.file(url,destfile="methylAnalysisDataV3.tar.gz"  method="auto")
}
*# extract the data*
if(!file.exists("./data")){
    untar("methylAnalysisDataV3.tar.gz",exdir=".",compressed="gzip")
}
                    


Once the data has been downloaded and extracted, there should be a folder called data that contains all the files necessary to execute the workflow.

                        # set up a path to the data directory

                        dataDirectory <- 
                        "./data"

                        # list the files

                        list.files
                        (dataDirectory, 
                        recursive=
                        TRUE
                        )
                    




                        ##  [1] "48639-non-specific-probes-Illumina450k.csv"
##  [2] "5975827018/5975827018_R06C02_Grn.idat"
##  [3] "5975827018/5975827018_R06C02_Red.idat"
##  [4] "6264509100/6264509100_R01C01_Grn.idat"
##  [5] "6264509100/6264509100_R01C01_Red.idat"
##  [6] "6264509100/6264509100_R01C02_Grn.idat"
##  [7] "6264509100/6264509100_R01C02_Red.idat"
##  [8] "6264509100/6264509100_R02C01_Grn.idat"
##  [9] "6264509100/6264509100_R02C01_Red.idat"
## [10] "6264509100/6264509100_R02C02_Grn.idat"
## [11] "6264509100/6264509100_R02C02_Red.idat"
## [12] "6264509100/6264509100_R03C01_Grn.idat"
## [13] "6264509100/6264509100_R03C01_Red.idat"
## [14] "6264509100/6264509100_R03C02_Grn.idat"
## [15] "6264509100/6264509100_R03C02_Red.idat"
## [16] "6264509100/6264509100_R04C01_Grn.idat"
## [17] "6264509100/6264509100_R04C01_Red.idat"
## [18] "6264509100/6264509100_R04C02_Grn.idat"
## [19] "6264509100/6264509100_R04C02_Red.idat"
## [20] "6264509100/6264509100_R05C01_Grn.idat"
## [21] "6264509100/6264509100_R05C01_Red.idat"
## [22] "6264509100/6264509100_R05C02_Grn.idat"
## [23] "6264509100/6264509100_R05C02_Red.idat"
## [24] "6264509100/6264509100_R06C01_Grn.idat"
## [25] "6264509100/6264509100_R06C01_Red.idat"
## [26] "6264509100/6264509100_R06C02_Grn.idat"
## [27] "6264509100/6264509100_R06C02_Red.idat"
## [28] "ageData.RData"
## [29] "human_c2_v5.rdata"
## [30] "model-based-cpg-islands-hg19-chr17.txt"
## [31] "SampleSheet.csv"
## [32] "wgEncodeRegDnaseClusteredV3chr17.bed"
                    


To demonstrate the various aspects of analysing methylation data, we will be using a small, publicly available 450k methylation dataset (GSE49667) ( Zhang *et al.*, 2013). The dataset contains 10 samples in total: there are 4 different sorted T-cell types (naive, rTreg, act_naive, act_rTreg, collected from 3 different individuals (M28, M29, M30). For details describing sample collection and preparation, see Zhang *et al.* (2013). An additional birth sample (individual VICS-72098-18-B) is included from another study (GSE51180) ( Cruickshank *et al.*, 2013) to illustrate approaches for identifying and excluding poor quality samples.

There are several R Bioconductor packages available that have been developed for analysing methylation array data, including *minfi* ( Aryee *et al.*, 2014), *missMethyl* ( Phipson *et al.*, 2016), *wateRmelon* ( Pidsley *et al.*, 2013), *methylumi* ( Davis *et al.*, 2015), *ChAMP* ( Morris *et al.*, 2014) and *charm* ( Aryee *et al.*, 2011). Some of the packages, such as *minfi* and *methylumi* include a framework for reading in the raw data from IDAT files and various specialised objects for storing and manipulating the data throughout the course of an analysis. Other packages provide specialised analysis methods for normalisation and statistical testing that rely on either *minfi* or *methylumi* objects. It is possible to convert between *minfi* and *methylumi* data types, however, this is not always trivial. Thus, it is advisable to consider the methods that you are interested in using and the data types that are most appropriate before you begin your analysis. Another popular method for analysing methylation array data is *limma* ( Ritchie *et al.*, 2015), which was originally developed for gene expression microarray analysis. As *limma* operates on a matrix of values, it is easily applied to any data that can be converted to a matrix in R. For a complete list of Bioconductor packages for analysing DNA methylation data, one can search for “DNAMethylation” in BiocViews ( https://www.bioconductor.org/packages/release/BiocViews.html#___DNAMethylation) on the Bioconductor website.

We will begin with an example of a **probe-wise** differential methylation analysis using *minfi* and *limma*. By **probe-wise** analysis we mean each individual CpG probe will be tested for differential methylation for the comparisons of interest and p-values and moderated t-statistics ( Smyth, 2004) will be generated for each CpG probe.

### Loading the data

It is useful to begin an analysis in R by loading all the packages that are likely to be required.



                        # load packages required for analysis

                        library
                        (limma)

                        library
                        (minfi)

                        library
                        (IlluminaHumanMethylation450kanno.ilmn12.hg19)

                        library
                        (IlluminaHumanMethylation450kmanifest)

                        library
                        (RColorBrewer)

                        library
                        (missMethyl)

                        library
                        (matrixStats)

                        library
                        (minfiData)

                        library
                        (Gviz)

                        library
                        (DMRcate)

                        library
                        (stringr)
                    


The *minfi*, *IlluminaHumanMethylation450kanno.ilmn12.hg19*, *IlluminaHumanMethylation450kmanifest*, *missMethyl*, *minfiData* and *DMRcate* are methylation specific packages, while *RColorBrewer* and *Gviz* are visualisation packages. We use *limma* for testing differential methylation, and *matrixStats* and *stringr* have functions used in the workflow. The *IlluminaHumanMethylation450kmanifest* package provides the Illumina manifest as an R object which can easily be loaded into the environment. The manifest contains all of the annotation information for each of the CpG probes on the 450k array. This is useful for determining where any differentially methylated probes are located in a genomic context.



                        # get the 450k annotation data

                        ann450k = 
                        getAnnotation
                        (IlluminaHumanMethylation450kanno.ilmn12.hg19)

                        head(ann450k)




                        ## DataFrame with 6 rows and 33 columns

                        ##		      chr	pos	 strand	       Name    AddressA

                        ##	      <character> <integer> <character> <character> <character>

                        ## cg00050873	     chrY   9363356	      -	 cg00050873    32735311

                        ## cg00212031	     chrY  21239348	      -	 cg00212031    29674443

                        ## cg00213748	     chrY   8148233	      -	 cg00213748    30703409

                        ## cg00214611	     chrY  15815688	      -  cg00214611    69792329

                        ## cg00455876	     chrY   9385539	      -  cg00455876    27653438

                        ## cg01707559	     chrY   6778695	      +  cg01707559    45652402

                        ##	         AddressB	                                   ProbeSeqA

                        ##	      <character>	                                 <character>

                        ## cg00050873	 31717405 ACAAAAAAACAACACACAACTATAATAATTTTTAAAATAAATAAACCCCA

                        ## cg00212031	 38703326 CCCAATTAACCACAAAAACTAAACAAATTATACAATCAAAAAAACATACA

                        ## cg00213748	 36767301 TTTTAACACCTAACACCATTTTAACAATAAAAATTCTACAAAAAAAAACA

                        ## cg00214611	 46723459 CTAACTTCCAAACCACACTTTATATACTAAACTACAATATAACACAAACA

                        ## cg00455876	 69732350 AACTCTAAACTACCCAACACAAACTCCAAAAACTTCTCAAAAAAAACTCA

                        ## cg01707559	 64689504 ACAAATTAAAAACACTAAAACAAACACAACAACTACAACAACAAAAAACA

                        ##	                                               ProbeSeqB	Type

                        ##	                                             <character> <character>

                        ## cg00050873 ACGAAAAAACAACGCACAACTATAATAATTTTTAAAATAAATAAACCCCG           I

                        ## cg00212031 CCCAATTAACCGCAAAAACTAAACAAATTATACGATCGAAAAAACGTACG           I

                        ## cg00213748 TTTTAACGCCTAACACCGTTTTAACGATAAAAATTCTACAAAAAAAAACG           I

                        ## cg00214611 CTAACTTCCGAACCGCGCTTTATATACTAAACTACAATATAACGCGAACG           I

                        ## cg00455876 AACTCTAAACTACCCGACACAAACTCCAAAAACTTCTCGAAAAAAACTCG           I

                        ## cg01707559 GCGAATTAAAAACACTAAAACGAACGCGACGACTACAACGACAAAAAACG           I

                        ##	         NextBase	Color	 Probe_rs Probe_maf	 CpG_rs

                        ##	      <character> <character> <character> <numeric> <character>

                        ## cg00050873	        A	  Red	       NA	 NA	     NA

                        ## cg00212031	        T	  Red	       NA	 NA	     NA

                        ## cg00213748	        A	  Red	       NA	 NA	     NA

                        ## cg00214611	        A	  Red	       NA	 NA	     NA

                        ## cg00455876	        A	  Red	       NA	 NA	     NA

                        ## cg01707559	        A	  Red	       NA	 NA	     NA

                        ##	        CpG_maf	     SBE_rs   SBE_maf           Islands_Name

                        ##	      <numeric> <character> <numeric>	         <character>

                        ## cg00050873	     NA	         NA	   NA   chrY:9363680-9363943

                        ## cg00212031	     NA	         NA	   NA chrY:21238448-21240005

                        ## cg00213748	     NA	         NA	   NA   chrY:8147877-8148210

                        ## cg00214611	     NA	         NA	   NA chrY:15815488-15815779

                        ## cg00455876	     NA	         NA	   NA   chrY:9385471-9385777

                        ## cg01707559	     NA	         NA	   NA   chrY:6778574-6780028

                        ##	      Relation_to_Island

                        ##	             <character>

                        ## cg00050873	         N_Shore

                        ## cg00212031	          Island

                        ## cg00213748	         S_Shore

                        ## cg00214611	          Island

                        ## cg00455876	          Island

                        ## cg01707559	          Island

                        ##

                        ##	      Forward_Sequence

                        ##	           <character>

                        ## cg00050873 TATCTCTGTCTGGCGAGGAGGCAACGCACAACTGTGGTGGTTTTTGGAGTGGGTGGACCC[CG]

                        ## cg00212031 CCATTGGCCCGCCCCAGTTGGCCGCAGGGACTGAGCAAGTTATGCGGTCGGGAAGACGTG[CG]

                        ## cg00213748 TCTGTGGGACCATTTTAACGCCTGGCACCGTTTTAACGATGGAGGTTCTGCAGGAGGGGG[CG]

                        ## cg00214611 GCGCCGGCAGGACTAGCTTCCGGGCCGCGCTTTGTGTGCTGGGCTGCAGTGTGGCGCGGG[CG]

                        ## cg00455876 CGCGTGTGCCTGGACTCTGAGCTACCCGGCACAAGCTCCAAGGGCTTCTCGGAGGAGGCT[CG]

                        ## cg01707559 AGCGGCCGCTCCCAGTGGTGGTCACCGCCAGTGCCAATCCCTTGCGCCGCCGTGCAGTCC[CG]

                        ##	                                               SourceSeq Random_Loci

                        ##	                                             <character> <character>

                        ## cg00050873 CGGGGTCCACCCACTCCAAAAACCACCACAGTTGTGCGTTGCCTCCTCGC

                        ## cg00212031 CGCACGTCTTCCCGACCGCATAACTTGCTCAGTCCCTGCGGCCAACTGGG

                        ## cg00213748 CGCCCCCTCCTGCAGAACCTCCATCGTTAAAACGGTGCCAGGCGTTAAAA

                        ## cg00214611 CGCCCGCGCCACACTGCAGCCCAGCACACAAAGCGCGGCCCGGAAGCTAG

                        ## cg00455876 GACTCTGAGCTACCCGGCACAAGCTCCAAGGGCTTCTCGGAGGAGGCTCG

                        ## cg01707559 CGCCCTCTGTCGCTGCAGCCGCCGCGCCCGCTCCAGTGCCCCCAATTCGC

                        ##	      Methyl27_Loci UCSC_RefGene_Name	     UCSC_RefGene_Accession

                        ##	        <character>       <character>	                <character>

                        ## cg00050873	               TSPY4;FAM197Y2	     NM_001164471;NR_001553

                        ## cg00212031	                       TTTY14	                  NR_001543

                        ## cg00213748

                        ## cg00214611	                TMSB4Y;TMSB4Y	        NM_004202;NM_004202

                        ## cg00455876

                        ## cg01707559	            TBL1Y;TBL1Y;TBL1Y NM_134259;NM_033284;NM_134258

                        ##	        UCSC_RefGene_Group     Phantom	       DMR    Enhancer

                        ##	               <character> <character> <character> <character>

                        ## cg00050873         Body;TSS1500

                        ## cg00212031	            TSS200

                        ## cg00213748

                        ## cg00214611	     1stExon;5'UTR

                        ## cg00455876

                        ## cg01707559 TSS200;TSS200;TSS200

                        ##	               HMM_Island Regulatory_Feature_Name

                        ##                    <character>	      <character>

                        ## cg00050873   Y:9973136-9976273

                        ## cg00212031 Y:19697854-19699393

                        ## cg00213748   Y:8207555-8208234

                        ## cg00214611 Y:14324883-14325218     Y:15815422-15815706

                        ## cg00455876   Y:9993394-9995882

                        ## cg01707559   Y:6838022-6839951

                        ##	                    Regulatory_Feature_Group	     DHS

                        ##	                                 <character> <character>

                        ## cg00050873

                        ## cg00212031

                        ## cg00213748

                        ## cg00214611 Promoter_Associated_Cell_type_specific

                        ## cg00455876

                        ## cg01707559
                    


As for their many other BeadArray platforms, Illumina methylation data is usually obtained in the form of Intensity Data (IDAT) Files. This is a proprietary format that is output by the scanner and stores summary intensities for each probe on the array. However, there are Bioconductor packages available that facilitate the import of data from IDAT files into R ( Smith *et al.*, 2013). Typically, each IDAT file is approximately 8MB in size. The simplest way to import the raw methylation data into R is using the *minfi* function read.metharray.sheet, along with the path to the IDAT files and a sample sheet. The sample sheet is a CSV (comma-separated) file containing one line per sample, with a number of columns describing each sample. The format expected by the read.metharray.sheet function is based on the sample sheet file that usually accompanies Illumina methylation array data. It is also very similar to the targets file described by the *limma* package. Importing the sample sheet into R creates a data.frame with one row for each sample and several columns. The read.metharray.sheet function uses the specified path and other information from the sample sheet to create a column called Basename which specifies the location of each individual IDAT file in the experiment.



                        # read in the sample sheet for the experiment

                        targets <- 
                        read.metharray.sheet
                        (dataDirectory, 
                        pattern=
                        "SampleSheet.csv"
                        )
                    




                        ## [read.metharray.sheet] Found the following CSV files:


                        ## [1] "./data/SampleSheet.csv"
                    




                        targets
                    




                        ##    Sample_Name  Sample_Well   Sample_Source Sample_Group Sample_Label

                        ## 1            1           A1             M28        naive        naive

                        ## 2            2           B1             M28        rTreg        rTreg

                        ## 3            3           C1             M28    act_naive    act_naive

                        ## 4            4           D1             M29        naive        naive

                        ## 5            5           E1             M29    act_naive    act_naive

                        ## 6            6           F1             M29    act_rTreg    act_rTreg

                        ## 7            7           G1             M30        naive        naive

                        ## 8            8           H1             M30        rTreg        rTreg

                        ## 9            9           A2             M30    act_naive    act_naive

                        ## 10          10           B2             M30    act_rTreg    act_rTreg

                        ## 11          11          H06 VICS-72098-18-B        birth        birth

                        ##      Pool_ID   Array      Slide                            Basename

                        ## 1 	   <NA>  R01C01 6264509100 ./data/6264509100/6264509100_R01C01

                        ## 2 	   <NA>  R02C01 6264509100 ./data/6264509100/6264509100_R02C01

                        ## 3 	   <NA>  R03C01 6264509100 ./data/6264509100/6264509100_R03C01

                        ## 4 	   <NA>  R04C01 6264509100 ./data/6264509100/6264509100_R04C01

                        ## 5 	   <NA>  R05C01 6264509100 ./data/6264509100/6264509100_R05C01

                        ## 6 	   <NA>  R06C01 6264509100 ./data/6264509100/6264509100_R06C01

                        ## 7 	   <NA>  R01C02 6264509100 ./data/6264509100/6264509100_R01C02

                        ## 8 	   <NA>  R02C02 6264509100 ./data/6264509100/6264509100_R02C02

                        ## 9 	   <NA>  R03C02 6264509100 ./data/6264509100/6264509100_R03C02

                        ## 10 	   <NA>  R04C02 6264509100 ./data/6264509100/6264509100_R04C02

                        ## 11 	   <NA>  R06C02 5975827018 ./data/5975827018/5975827018_R06C02
                    


Now that we have imported the information about the samples and where the data is located, we can read the raw intensity signals into R from the IDAT files using the read.metharray.exp function. This creates an RGChannelSet object that contains all the raw intensity data, from both the red and green colour channels, for each of the samples. At this stage, it can be useful to rename the samples with more descriptive names.



                        # read in the raw data from the IDAT files

                        rgSet <- 
                        read.metharray.exp(
                        targets=
                        targets)
rgSet
                    




                        ## RGChannelSet (storageMode: lockedEnvironment)
## assayData: 622399 features, 11 samples
##   element names: Green, Red
## An object of class 'AnnotatedDataFrame'
##   sampleNames: 6264509100_R01C01 6264509100_R02C01 ...
##     5975827018_R06C02 (11 total)
##   varLabels: Sample_Name Sample_Well ... filenames (10 total)
##   varMetadata: labelDescription
## Annotation
##   array: IlluminaHumanMethylation450k
##   annotation: ilmn12.hg19
                    





                        # give the samples descriptive names

                        targets$ID <- 
                        paste
                        (targets$Sample_Group,targets$Sample_Name,
                        sep=
                        "."
                        )

                        sampleNames
                        (rgSet) <- targets$ID

                        rgSet
                    





                        ## RGChannelSet (storageMode: lockedEnvironment)
## assayData: 622399 features, 11 samples
##   element names: Green, Red
## An object of class 'AnnotatedDataFrame'
##   sampleNames: naive.1 rTreg.2 ... birth.11 (11 total)
##   varLabels: Sample_Name Sample_Well ... filenames (10 total)
##   varMetadata: labelDescription
## Annotation
##   array: IlluminaHumanMethylation450k
##   annotation: ilmn12.hg19
                    


### Quality control

Once the data has been imported into R, we can evaluate its quality. Firstly, we need to calculate detection p-values. We can generate a detection p-value for every CpG in every sample, which is indicative of the quality of the signal. The method used by *minfi* to calculate detection p-values compares the total signal ( *M* + *U*) for each probe to the background signal level, which is estimated from the negative control probes. Very small p-values are indicative of a reliable signal whilst large p-values, for example >0.01, generally indicate a poor quality signal.

**Figure 2.**
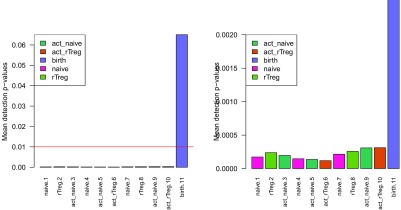
Mean detection p-values summarise the quality of the signal across all the probes in each sample. The plot on the right is a zoomed in version of the plot on the left.

Plotting the mean detection p-value for each sample allows us to gauge the general quality of the samples in terms of the overall signal reliability ( Figure 2). Samples that have many failed probes will have relatively large mean detection p-values.




                        # calculate the detection p-values

                        detP <- 
                        detectionP
                        (rgSet)

                        head
                        (detP)
                    





                        
##            naive.1 rTreg.2  act_naive.3 naive.4 act_naive.5 act_rTreg.6
## cg00050873       0       0 0.000000e+00       0 0.00000e+00           0
## cg00212031       0       0 0.000000e+00       0 0.00000e+00           0
## cg00213748       0	    0 1.181832e-12       0 8.21565e-15           0
## cg00214611       0       0 0.000000e+00       0 0.00000e+00           0
## cg00455876       0       0 0.000000e+00       0 0.00000e+00           0
## cg01707559       0       0 0.000000e+00       0 0.00000e+00           0
##            naive.7      rTreg.8 act_naive.9 act_rTreg.10  birth.11
## cg00050873       0 0.000000e+00           0 0.000000e+00 0.0000000
## cg00212031       0 0.000000e+00           0 0.000000e+00 0.0000000
## cg00213748       0 1.469801e-05           0 1.365951e-08 0.6735224
## cg00214611       0 0.000000e+00           0 0.000000e+00 0.7344451
## cg00455876       0 0.000000e+00           0 0.000000e+00 0.0000000
## cg01707559       0 0.000000e+00           0 0.000000e+00 0.0000000
                    





                        # examine mean detection p-values across all samples to identify any failed samples

                        pal <- 
                        brewer.pal
                        (
                        8
                        ,
                        "Dark2"
                        )

                        par
                        (
                        mfrow=c
                        (
                        1
                        ,
                        2
                        ))

                        barplot
                        (
                        colMeans
                        (detP), 
                        col=
                        pal[
                        factor
                        (targets$Sample_Group)], 
                        las=
                        2
                        ,
         
                        cex.names=
                        0.8
                        ,
                        ylab=
                        "Mean detection p-values"
                        )

                        abline
                        (
                        h=
                        0.01
                        ,
                        col=
                        "red"
                        )

                        legend
                        (
                        "topleft"
                        , 
                        legend=levels
                        (
                        factor
                        (targets$Sample_Group)), 
                        fill=
                        pal,
        
                        bg=
                        "white"
                        )


                        barplot
                        (
                        colMeans
                        (detP), 
                        col=
                        pal[
                        factor
                        (targets$Sample_Group)], 
                        las=
                        2
                        ,
         
                        cex.names=
                        0.8
                        , 
                        ylim = c
                        (
                        0
                        ,
                        0.002
                        ), 
                        ylab=
                        "Mean detection p-values"
                        )

                        legend
                        (
                        "topleft"
                        , 
                        legend=levels
                        (
                        factor
                        (targets$Sample_Group)), 
                        fill=
                        pal,
        
                        bg=
                        "white"
                        )
                    


The *minfi* qcReport function generates many other useful quality control plots. The *minfi* vignette describes the various plots and how they should be interpreted in detail. Generally, samples that look poor based on mean detection p-value will also look poor using other metrics and it is usually advisable to exclude them from further analysis.



                        qcReport
                        (rgSet, 
                        sampNames=
                        targets$ID, 
                        sampGroups=
                        targets$Sample_Group,
          
                        pdf=
                        "qcReport.pdf"
                        )
                    


Poor quality samples can be easily excluded from the analysis using a detection p-value cutoff, for example >0.05. For this particular dataset, the birth sample shows a very high mean detection p-value, and hence it is excluded from subsequent analysis ( Figure 2).



                        # remove poor quality samples

                        keep <- 
                        colMeans
                        (detP) < 
                        0.05

                        rgSet <- rgSet[,keep]

                        rgSet
                    




                        ## RGChannelSet (storageMode: lockedEnvironment)
## assayData: 622399 features, 10 samples
##   element names: Green, Red
## An object of class 'AnnotatedDataFrame'
##   sampleNames: naive.1 rTreg.2 ... act_rTreg.10 (10 total)
##   varLabels: Sample_Name Sample_Well ... filenames (10 total)
##   varMetadata: labelDescription
## Annotation
##   array: IlluminaHumanMethylation450k
##   annotation: ilmn12.hg19
                    




                        # remove poor quality samples from targets data

                        targets <- targets[keep,]

                        targets[,
                        1:
                        5
                        ]
                    




                        
##    Sample_Name Sample_Well Sample_Source Sample_Group Sample_Label
## 1	        1          A1           M28        naive        naive
## 2            2          B1           M28        rTreg	rTreg
## 3            3          C1           M28    act_naive    act_naive
## 4            4          D1           M29        naive        naive
## 5            5          E1           M29    act_naive    act_naive
## 6            6          F1           M29    act_rTreg    act_rTreg
## 7            7          G1           M30        naive        naive
## 8            8          H1           M30        rTreg        rTreg
## 9            9          A2           M30    act_naive    act_naive
## 10          10          B2           M30    act_rTreg    act_rTreg
                    




                        # remove poor quality samples from detection p-value table

                        detP <- detP[,keep]

                        dim
                        (detP)
                    




                        ## [1] 485512  10
                    


### Normalisation

**Figure 3.**
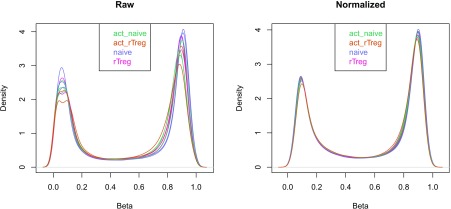
The density plots show the distribution of the beta values for each sample before and after normalisation.

To minimise the unwanted variation within and between samples, various data normalisations can be applied. Many different types of normalisation have been developed for methylation arrays and it is beyond the scope of this workflow to compare and contrast all of them ( Fortin *et al.*, 2014; Maksimovic *et al.*, 2012; Mancuso *et al.*, 2011; Pidsley *et al.*, 2013; Sun *et al.*, 2011; Teschendorff *et al.*, 2013; Touleimat & Tost, 2012; Triche *et al.*, 2013; Wang *et al.*, 2012; Wu *et al.*, 2014). Several methods have been built into *minfi* and can be directly applied within its framework ( Fortin *et al.*, 2014; Maksimovic *et al.*, 2012; Triche *et al.*, 2013; Touleimat & Tost, 2012), whilst others are *methylumi*-specific or require custom data types ( Mancuso *et al.*, 2011; Pidsley *et al.*, 2013; Sun *et al.*, 2011; Teschendorff *et al.*, 2013; Wang *et al.*, 2012; Wu *et al.*, 2014). Although there is no single normalisation method that is universally considered best, a recent study by Fortin *et al.* (2014) has suggested that a good rule of thumb within the *minfi* framework is that the preprocessFunnorm ( Fortin *et al.*, 2014) function is most appropriate for datasets with global methylation differences such as cancer/normal or vastly different tissue types, whilst the preprocessQuantile function ( Touleimat & Tost, 2012) is more suited for datasets where you do not expect global differences between your samples, for example a single tissue. Further discussion on appropriate choice of normalisation can be found in ( Hicks & Irizarry, 2015), and the accompanying *quantro* package includes data-driven tests for the assumptions of quantile normalisation. As we are comparing different blood cell types, which are globally relatively similar, we will apply the preprocessQuantile method to our data ( Figure 3). This function implements a stratified quantile normalisation procedure which is applied to the methylated and unmethylated signal intensities separately, and takes into account the different probe types. Note that after normalisation, the data is housed in a GenomicRatioSet object. This is a much more compact representation of the data as the colour channel information has been discarded and the *M* and *U* intensity information has been converted to M-values and beta values, together with associated genomic coordinates. Note, running the preprocessQuantile function on this dataset produces the warning: ‘An inconsistency was encountered while determining sex’; this can be ignored as it is due to all the samples being from male donors.




                        # normalize the data; this results in a GenomicRatioSet object

                        mSetSq <- 
                        preprocessQuantile
                        (rgSet)
                    





                        
## [preprocessQuantile] Mapping to genome.

## [preprocessQuantile] Fixing outliers.

## Warning in .getSex(CN = CN, xIndex = xIndex, yIndex = yIndex, cutoff
## = cutoff): An inconsistency was encountered while determining sex. One
## possibility is that only one sex is present. We recommend further checks,
## for example with the plotSex function.

## [preprocessQuantile] Quantile normalizing.
                    





                        # create a MethylSet object from the raw data for plotting

                        mSetRaw <- 
                        preprocessRaw
                        (rgSet)
                    





                        # visualise what the data looks like before and after normalisation

                        par
                        (
                        mfrow=c
                        (
                        1
                        ,
                        2
                        ))

                        densityPlot
                        (rgSet, 
                        sampGroups=
                        targets$Sample_Group,
                        main=
                        "Raw"
                        , 
                        legend=
                        FALSE
                        )

                        legend
                        (
                        "top"
                        , 
                        legend = levels
                        (
                        factor
                        (targets$Sample_Group)),
        
                        text.col=brewer.pal
                        (
                        8
                        ,
                        "Dark2"
                        ))

                        densityPlot
                        (
                        getBeta
                        (mSetSq), 
                        sampGroups=
                        targets$Sample_Group,
              
                        main=
                        "Normalized"
                        , 
                        legend=
                        FALSE
                        )

                        legend
                        (
                        "top"
                        , 
                        legend = levels
                        (
                        factor
                        (targets$Sample_Group)),
        
                        text.col=brewer.pal
                        (
                        8
                        ,
                        "Dark2"
                        ))
                    


### Data exploration

Multi-dimensional scaling (MDS) plots are excellent for visualising data, and are usually some of the first plots that should be made when exploring the data. MDS plots are based on principal components analysis and are an unsupervised method for looking at the similarities and differences between the various samples. Samples that are more similar to each other should cluster together, and samples that are very different should be further apart on the plot. Dimension one (or principal component one) captures the greatest source of variation in the data, dimension two captures the second greatest source of variation in the data and so on. Colouring the data points or labels by known factors of interest can often highlight exactly what the greatest sources of variation are in the data. It is also possible to use MDS plots to decipher sample mix-ups.



                        # MDS plots to look at largest sources of variation

                        par
                        (
                        mfrow=c
                        (
                        1
                        ,
                        2
                        ))

                        plotMDS
                        (
                        getM
                        (mSetSq), 
                        top=
                        1000
                        , 
                        gene.selection=
                        "common"
                        ,
         
                        col=
                        pal[
                        factor
                        (targets$Sample_Group)])

                        legend
                        (
                        "top"
                        , 
                        legend=levels
                        (
                        factor
                        (targets$Sample_Group)), 
                        text.col=
                        pal,
        
                        bg=
                        "white"
                        , 
                        cex=
                        0.7
                        )


                        plotMDS
                        (
                        getM
                        (mSetSq), 
                        top=
                        1000
                        , 
                        gene.selection=
                        "common"
                        ,
         
                        col=
                        pal[
                        factor
                        (targets$Sample_Source)])

                        legend
                        (
                        "top"
                        , 
                        legend=levels
                        (
                        factor
                        (targets$Sample_Source)), 
                        text.col=
                        pal,
        
                        bg=
                        "white"
                        , 
                        cex=
                        0.7
                        )
                    


**Figure 4.**
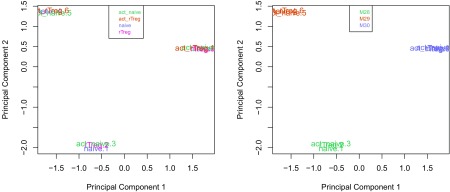
Multi-dimensional scaling plots are a good way to visualise the relationships between the samples in an experiment.



**Figure 5.**
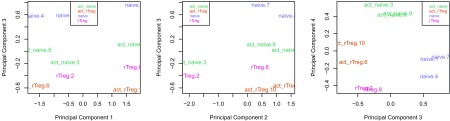
Examining the higher dimensions of an MDS plot can reaveal significant sources of variation in the data.

Examining the MDS plots for this dataset demonstrates that the largest source of variation is the difference between individuals ( Figure 4). The higher dimensions reveal that the differences between cell types are largely captured by the third and fourth principal components ( Figure 5). This type of information is useful in that it can inform downstream analysis. If obvious sources of unwanted variation are revealed by the MDS plots, we can include them in our statistical model to account for them. In the case of this particular dataset, we will include individual to individual variation in our statistical model.

                        # Examine higher dimensions to look at other sources of variation

                        par
                        (
                        mfrow=c
                        (
                        1
                        ,
                        3
                        ))

                        plotMDS
                        (
                        getM
                        (mSetSq), 
                        top=
                        1000
                        , 
                        gene.selection=
                        "common"
                        ,
         
                        col=
                        pal[
                        factor
                        (targets$Sample_Group)], 
                        dim=c
                        (
                        1
                        ,
                        3
                        ))

                        legend
                        (
                        "top"
                        , 
                        legend=levels
                        (
                        factor
                        (targets$Sample_Group)), 
                        text.col=
                        pal,
        
                        cex=
                        0.7
                        , 
                        bg=
                        "white"
                        )


                        plotMDS
                        (
                        getM
                        (mSetSq), 
                        top=
                        1000
                        , 
                        gene.selection=
                        "common"
                        ,
         
                        col=
                        pal[
                        factor
                        (targets$Sample_Group)], 
                        dim=c
                        (
                        2
                        ,
                        3
                        ))

                        legend
                        (
                        "topleft"
                        , 
                        legend=levels
                        (
                        factor
                        (targets$Sample_Group)), 
                        text.col=
                        pal,
        
                        cex=
                        0.7
                        , 
                        bg=
                        "white"
                        )


                        plotMDS
                        (
                        getM
                        (mSetSq), 
                        top=
                        1000
                        , 
                        gene.selection=
                        "common"
                        ,
         
                        col=
                        pal[
                        factor
                        (targets$Sample_Group)], 
                        dim=c
                        (
                        3
                        ,
                        4
                        ))

                        legend
                        (
                        "topright"
                        , 
                        legend=levels
                        (
                        factor
                        (targets$Sample_Group)), 
                        text.col=
                        pal,
        
                        cex=
                        0.7
                        , 
                        bg=
                        "white"
                        )
                    


### Filtering

Poor performing probes are generally filtered out prior to differential methylation analysis. As the signal from these probes is unreliable, by removing them we perform fewer statistical tests and thus incur a reduced multiple testing penalty. We filter out probes that have failed in one or more samples based on detection p-value.



                        # ensure probes are in the same order in the mSetSq and detP objects

                        detP <- detP[
                        match
                        (
                        featureNames
                        (mSetSq),
                        rownames
                        (detP)),]


                        # remove any probes that have failed in one or more samples

                        keep <- 
                        rowSums
                        (detP < 
                        0.01
                        ) == 
                        ncol
                        (mSetSq)

                        table
                        (keep)
                    




                        ## keep
##  FALSE    TRUE
##    977  484535
                    




                        mSetSqFlt <- mSetSq[keep,]
mSetSqFlt
                    




                        ## class: GenomicRatioSet
## dim: 484535 10
## metadata(0):
## assays(2): M CN
## rownames(484535): cg13869341 cg14008030 ... cg08265308 cg14273923
## rowData names(0):
## colnames(10): naive.1 rTreg.2 ... act_naive.9 act_rTreg.10
## colData names(11): Sample_Name Sample_Well ... filenames
##   predictedSex
## Annotation
##   array: IlluminaHumanMethylation450k
##   annotation: ilmn12.hg19
## Preprocessing
##   Method: Raw (no normalization or bg correction)
##   minfi version: 1.20.2
##   Manifest version: 0.4.0
                    


Depending on the nature of your samples and your biological question you may also choose to filter out the probes from the X and Y chromosomes or probes that are known to have common SNPs at the CpG site. As the samples in this dataset were all derived from male donors, we will not be removing the sex chromosome probes as part of this analysis, however example code is provided below. A different dataset, which contains both male and female samples, is used to demonstrate a Differential Variability analysis and provides an example of when sex chromosome removal is necessary ( Figure 13).



                        # if your data includes males and females, remove probes on the sex chromosomes

                        keep <- !(
                        featureNames
                        (mSetSqFlt) %in% ann450k$Name[ann450k$chr %in%
                                                               
                        c
                        (
                        "chrX"
                        ,
                        "chrY"
                        )])

                        table
                        (keep)
mSetSqFlt <- mSetSqFlt[keep,]
                    


There is a function in *minfi* that provides a simple interface for the removal of probes where common SNPs may affect the CpG. You can either remove all probes affected by SNPs (default), or only those with minor allele frequencies greater than a specified value.



                        # remove probes with SNPs at CpG site

                        mSetSqFlt <- 
                        dropLociWithSnps
                        (mSetSqFlt)
mSetSqFlt
                    




                        ## class: GenomicRatioSet
## dim: 467351 10
## metadata(0):
## assays(2): M CN
## rownames(467351): cg13869341 cg14008030 ... cg08265308 cg14273923
## rowData names(0):
## colnames(10): naive.1 rTreg.2 ... act_naive.9 act_rTreg.10
## colData names(11): Sample_Name Sample_Well ... filenames
##   predictedSex
## Annotation
##   array: IlluminaHumanMethylation450k
##   annotation: ilmn12.hg19
## Preprocessing
##   Method: Raw (no normalization or bg correction)
##   minfi version: 1.20.2
##   Manifest version: 0.4.0
                    


We will also filter out probes that have shown to be cross-reactive, that is, probes that have been demonstrated to map to multiple places in the genome. This list was originally published by Chen *et al.* (2013) and can be obtained from the authors’ website.



                        # exclude cross reactive probes

                        xReactiveProbes <- 
                        read.csv
                        (
                        file=paste
                        (dataDirectory,
                               
                        "48639-non-specific-probes-Illumina450k.csv"
                        ,
                               
                        sep=
                        "/"
                        ), 
                        stringsAsFactors=
                        FALSE
                        )
keep <- !(
                        featureNames
                        (mSetSqFlt) %in% xReactiveProbes$TargetID)

                        table
                        (keep)
                    




                        ## keep
##  FALSE   TRUE
##  27433 439918
                    




                        mSetSqFlt <- mSetSqFlt[keep,]
mSetSqFlt
                    




                        ## class: GenomicRatioSet
## dim: 439918 10
## metadata(0):
## assays(2): M CN
## rownames(439918): cg13869341 cg24669183 ... cg08265308 cg14273923
## rowData names(0):
## colnames(10): naive.1 rTreg.2 ... act_naive.9 act_rTreg.10
## colData names(11): Sample_Name Sample_Well ... filenames
##   predictedSex
## Annotation
##   array: IlluminaHumanMethylation450k
##   annotation: ilmn12.hg19
## Preprocessing
##   Method: Raw (no normalization or bg correction)
##   minfi version: 1.20.2
##   Manifest version: 0.4.0
                    


**Figure 6.**
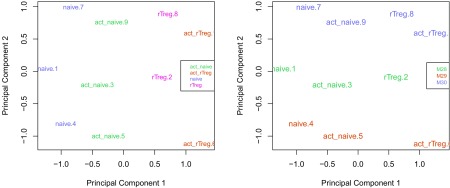
Removing SNP-affected CpGs probes from the data changes the sample clustering in the MDS plots.

**Figure 7.**
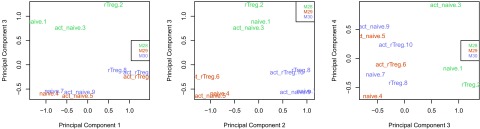
Examining the higher dimensions of the MDS plots shows that significant inter-individual variation still exists in the second and third principal components.

Once the data has been filtered and normalised, it is often useful to re-examine the MDS plots to see if the relationship between the samples has changed. It is apparent from the new MDS plots that much of the inter-individual variation has been removed as this is no longer the first principal component ( Figure 6), likely due to the removal of the SNP-affected CpG probes. However, the samples do still cluster by individual in the second dimension ( Figure 6 and Figure 7) and thus a factor for individual should still be included in the model.



                        par
                        (
                        mfrow=c
                        (
                        1
                        ,
                        2
                        ))

                        plotMDS
                        (
                        getM
                        (mSetSqFlt), 
                        top=
                        1000
                        , 
                        gene.selection=
                        "common"
                        ,
         
                        col=
                        pal[
                        factor
                        (targets$Sample_Group)], 
                        cex=
                        0.8
                        )

                        legend
                        (
                        "right"
                        , 
                        legend=levels
                        (
                        factor
                        (targets$Sample_Group)), 
                        text.col=
                        pal,
        
                        cex=
                        0.65
                        , 
                        bg=
                        "white"
                        )


                        plotMDS
                        (
                        getM
                        (mSetSqFlt), 
                        top=
                        1000
                        , 
                        gene.selection=
                        "common"
                        ,
         
                        col=
                        pal[
                        factor
                        (targets$Sample_Source)])

                        legend
                        (
                        "right"
                        , 
                        legend=levels
                        (
                        factor
                        (targets$Sample_Source)), 
                        text.col=
                        pal,
        
                        cex=
                        0.7
                        , 
                        bg=
                        "white"
                        )
                    




                        par
                        (
                        mfrow=c
                        (
                        1
                        ,
                        3
                        ))
        
                        # Examine higher dimensions to look at other sources of variation

                        plotMDS
                        (
                        getM
                        (mSetSqFlt), 
                        top=
                        1000
                        , 
                        gene.selection=
                        "common"
                        ,
         
                        col=
                        pal[
                        factor
                        (targets$Sample_Source)], 
                        dim=c
                        (
                        1
                        ,
                        3
                        ))

                        legend
                        (
                        "right"
                        , 
                        legend=levels
                        (
                        factor
                        (targets$Sample_Source)), 
                        text.col=
                        pal,
        
                        cex=
                        0.7
                        , 
                        bg=
                        "white"
                        )


                        plotMDS
                        (
                        getM
                        (mSetSqFlt), 
                        top=
                        1000
                        , 
                        gene.selection=
                        "common"
                        ,
         
                        col=
                        pal[
                        factor
                        (targets$Sample_Source)], 
                        dim=c
                        (
                        2
                        ,
                        3
                        ))

                        legend
                        (
                        "topright"
                        , 
                        legend=levels
                        (
                        factor
                        (targets$Sample_Source)), 
                        text.col=
                        pal,
        
                        cex=
                        0.7
                        , 
                        bg=
                        "white"
                        )


                        plotMDS
                        (
                        getM
                        (mSetSqFlt), 
                        top=
                        1000
                        , 
                        gene.selection=
                        "common"
                        ,
         
                        col=
                        pal[
                        factor
                        (targets$Sample_Source)], 
                        dim=c
                        (
                        3
                        ,
                        4
                        ))

                        legend
                        (
                        "right"
                        , 
                        legend=levels
                        (
                        factor
                        (targets$Sample_Source)), 
                        text.col=
                        pal,
        
                        cex=
                        0.7
                        , 
                        bg=
                        "white"
                        )
                    


**Figure 8.**
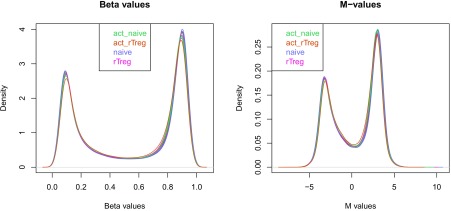
The distributions of beta and M-values are quite different; beta values are constrained between 0 and 1 whilst M-values range between -Inf and Inf.

The next step is to calculate M-values and beta values ( Figure 8). As previously mentioned, M-values have nicer statistical properties and are thus better for use in statistical analysis of methylation data whilst beta values are easy to interpret and are thus better for displaying data. A detailed comparison of M-values and beta values was published by Du *et al.* (2010).



                        # calculate M-values for statistical analysis

                        mVals <- 
                        getM
                        (mSetSqFlt)

                        head
                        (mVals[,
                        1
                        :
                        5
                        ])
                    




                        ##              naive.1   rTreg.2 act_naive.3   naive.4  act_naive.5
## cg13869341  2.421276  2.515948    2.165745  2.286314     2.109441
## cg24669183  2.169414  2.235964    2.280734  1.632309     2.184435
## cg15560884  1.761176  1.577578    1.597503  1.777486     1.764999
## cg01014490 -3.504268 -3.825119   -5.384735 -4.537864    -4.296526
## cg17505339  3.082191  3.924931    4.163206  3.255373     3.654134
## cg11954957  1.546401  1.912204    1.727910  2.441267     1.618331
                    




                        bVals <- 
                        getBeta
                        (mSetSqFlt)

                        head
                        (bVals[,
                        1
                        :
                        5
                        ])
                    




                        ##               naive.1    rTreg.2 act_naive.3    naive.4 act_naive.5
## cg13869341 0.84267937 0.85118462   0.8177504 0.82987650  0.81186174
## cg24669183 0.81812908 0.82489238   0.8293297 0.75610281  0.81967323
## cg15560884 0.77219626 0.74903910   0.7516263 0.77417882  0.77266205
## cg01014490 0.08098986 0.06590459   0.0233755 0.04127262  0.04842397
## cg17505339 0.89439216 0.93822870   0.9471357 0.90520570  0.92641305
## cg11954957 0.74495496 0.79008516   0.7681146 0.84450764  0.75431167
                    




                        par
                        (
                        mfrow=c
                        (
                        1
                        ,
                        2
                        ))

                        densityPlot
                        (bVals, 
                        sampGroups=
                        targets$Sample_Group, 
                        main=
                        "Beta values"
                        ,
             
                        legend=
                        FALSE
                        , 
                        xlab=
                        "Beta values"
                        )

                        legend
                        (
                        "top"
                        , 
                        legend = levels
                        (
                        factor
                        (targets$Sample_Group)),
        
                        text.col=brewer.pal
                        (
                        8
                        ,
                        "Dark2"
                        ))

                        densityPlot
                        (mVals, 
                        sampGroups=
                        targets$Sample_Group, 
                        main=
                        "M-values"
                        ,
             
                        legend=
                        FALSE
                        , 
                        xlab=
                        "M values"
                        )

                        legend
                        (
                        "topleft"
                        , 
                        legend = levels
                        (
                        factor
                        (targets$Sample_Group)),
        
                        text.col=brewer.pal
                        (
                        8
                        ,
                        "Dark2"
                        ))
                    


### Probe-wise differential methylation analysis

The biological question of interest for this particular dataset is to discover differentially methylated probes between the different cell types. However, as was apparent in the MDS plots, there is another factor that we need to take into account when we perform the statistical analysis. In the targets file, there is a column called Sample_Source, which refers to the individuals that the samples were collected from. In this dataset, each of the individuals contributes more than one cell type. For example, individual M28 contributes naive, rTreg and act_naive samples. Hence, when we specify our design matrix, we need to include two factors: individual and cell type. This style of analysis is called a paired analysis; differences between cell types are calculated *within* each individual, and then these differences are averaged *across* individuals to determine whether there is an overall significant difference in the mean methylation level for each CpG site. The *limma* User’s Guide extensively covers the different types of designs that are commonly used for microarray experiments and how to analyse them in R.

We are interested in pairwise comparisons between the four cell types, taking into account individual to individual variation. We perform this analysis on the matrix of M-values in *limma*, obtaining moderated t-statistics and associated p-values for each CpG site. A convenient way to set up the model when the user has many comparisons of interest that they would like to test is to use a contrasts matrix in conjunction with the design matrix. A contrasts matrix will take linear combinations of the columns of the design matrix corresponding to the comparisons of interest.

Since we are performing hundreds of thousands of hypothesis tests, we need to adjust the p-values for multiple testing. A common procedure for assessing how statistically significant a change in mean levels is between two groups when a very large number of tests is being performed is to assign a cut-off on the false discovery rate ( Benjamini & Hochberg, 1995), rather than on the unadjusted p-value. Typically 5% FDR is used, and this is interpreted as the researcher willing to accept that from the list of significant differentially methylated CpG sites, 5% will be false discoveries. If the p-values are not adjusted for multiple testing, the number of false discoveries will be unacceptably high. For this dataset, assuming a Type I error rate of 5%, we would expect to see 0.05*439918=21996 statistical significant results for a given comparison, even if there were truly no differentially methylated CpG sites.

Based on a false discovery rate of 5%, there are 3021 significantly differentially methylated CpGs in the naïve vs rTreg comparison, while rTreg vs act_rTreg doesn’t show any significant differential methylation.



                        # this is the factor of interest

                        cellType <- 
                        factor
                        (targets$Sample_Group)

                        # this is the individual effect that we need to account for

                        individual <- 
                        factor
                        (targets$Sample_Source)


                        # use the above to create a design matrix

                        design <- 
                        model.matrix
                        (~
                        0
                        +cellType+individual, 
                        data=
                        targets)

                        colnames
                        (design) <- 
                        c
                        (
                        levels
                        (cellType),
                        levels
                        (individual)[-
                        1
                        ])


                        # fit the linear model

                        fit <- 
                        lmFit
                        (mVals, design)

                        # create a contrast matrix for specific comparisons

                        contMatrix <- 
                        makeContrasts
                        (naive-rTreg,
			   naive-act_naive,
			   rTreg-act_rTreg,
			   act_naive-act_rTreg,
			      
                        levels=
                        design)
contMatrix
                    




                        ## 	     Contrasts
## Levels     naive - rTreg naive - act_naive rTreg - act_rTreg
##   act_naive 		  0 		   -1 		      0
##   act_rTreg 		  0 		    0 		     -1
##   naive 		  1 		    1 		      0
##   rTreg 		 -1 		    0 		      1
##   M29 		  0 		    0 		      0
##   M30 		  0 		    0 		      0
## 	     Contrasts
## Levels     act_naive - act_rTreg
##   act_naive 		  	  1
##   act_rTreg 		  	 -1
##   naive 		  	  0
##   rTreg 		  	  0
##   M29 		  	  0
##   M30 		  	  0
                    




                        # fit the contrasts

                        fit2 <- 
                        contrasts.fit
                        (fit, contMatrix)
fit2 <- 
                        eBayes
                        (fit2)


                        # look at the numbers of DM CpGs at FDR < 0.05

                        summary
                        (
                        decideTests
                        (fit2))
                    




                        ##     naive - rTreg naive - act_naive rTreg - act_rTreg act_naive - act_rTreg
## -1 		1618 		   400 		       0 		   559
## 0 	      436897 		439291 		  439918 	        438440
## 1 		1403 		   227 		       0 		   919
                    


We can extract the tables of differentially expressed CpGs for each comparison, ordered by B-statistic by default, using the topTable function in *limma*. The B-statistic is the log-odds of differential methylation, first published by Lonnstedt and Speed ( Lonnstedt & Speed, 2002). To order by p-value, the user can specify sort.by="p"; and in most cases, the ordering based on the p-value and ordering based on the B-statistic will be identical. The results of the analysis for the first comparison, naive vs. rTreg, can be saved as a data.frame by setting coef=1. The coef parameter explicitly refers to the column in the contrasts matrix which corresponds to the comparison of interest.



                        # get the table of results for the first contrast (naive - rTreg)

                        ann450kSub <- ann450k[
                        match
                        (
                        rownames
                        (mVals),ann450k$Name),
                         
                        c
                        (
                        1
                        :
                        4
                        ,
                        12
                        :
                        19
                        ,
                        24
                        :
                        ncol
                        (ann450k))]
DMPs <- 
                        topTable
                        (fit2,  
                        num=
                        Inf
                        , 
                        coef=
                        1
                        , 
                        genelist=
                        ann450kSub)

                        head
                        (DMPs)
                    




                        ## 		chr 	  pos strand 	   Name Probe_rs Probe_maf CpG_rs
## cg07499259  chr1  12188502 	   + cg07499259     <NA> 	NA   <NA>
## cg26992245  chr8  29848579 	   - cg26992245     <NA> 	NA   <NA>
## cg09747445 chr15  70387268 	   - cg09747445     <NA> 	NA   <NA>
## cg18808929  chr8  61825469 	   - cg18808929     <NA> 	NA   <NA>
## cg25015733  chr2  99342986 	   - cg25015733     <NA> 	NA   <NA>
## cg21179654  chr3 114057297 	   + cg21179654     <NA> 	NA   <NA>
## 	      CpG_maf SBE_rs SBE_maf 		Islands_Name
## cg07499259 	   NA   <NA> 	  NA
## cg26992245 	   NA   <NA> 	  NA
## cg09747445 	   NA   <NA> 	  NA chr15:70387929-70393206
## cg18808929 	   NA   <NA> 	  NA  chr8:61822358-61823028
## cg25015733 	   NA   <NA> 	  NA  chr2:99346882-99348177
## cg21179654 	   NA   <NA> 	  NA
## 	      Relation_to_Island
## cg07499259 		 OpenSea
## cg26992245 		 OpenSea
## cg09747445 		 N_Shore
## cg18808929 		 S_Shelf
## cg25015733 		 N_Shelf
## cg21179654 		 OpenSea
## 					     UCSC_RefGene_Name
## cg07499259 				       TNFRSF8;TNFRSF8
## cg26992245
## cg09747445 				        TLE3;TLE3;TLE3
## cg18808929
## cg25015733 				          	MGAT4A
## cg21179654 ZBTB20;ZBTB20;ZBTB20;ZBTB20;ZBTB20;ZBTB20;ZBTB20
## 									       
## 									      
##   UCSC_RefGene_Accession								      
## cg07499259 					        NM_152942;NM_001243
## cg26992245
## cg09747445 							    
## cg18808929
## cg25015733 										    
## cg21179654 NM_001164343;NM_001164346;NM_001164345;NM_001164342;
## 				     UCSC_RefGene_Group Phantom DMR Enhancer
## cg07499259 				     5'UTR;Body
## cg26992245 								TRUE
## cg09747445			         Body;Body;Body
## cg18808929 								TRUE
## cg25015733 				          5'UTR
## cg21179654 3'UTR;3'UTR;3'UTR;3'UTR;3'UTR;3'UTR;3'UTR
## 		       HMM_Island Regulatory_Feature_Name
## cg07499259 1:12111023-12111225
## cg26992245
## cg09747445
## cg18808929
## cg25015733
## cg21179654 			    3:114057192-114057775
## 		     Regulatory_Feature_Group DHS     logFC     AveExpr
## cg07499259 					   3.654104  2.46652171
## cg26992245 					   4.450696 -0.09180715
## cg09747445 					  -3.337299 -0.25201484
## cg18808929 					  -2.990263  0.77522878
## cg25015733 					  -3.054336  0.83280190
## cg21179654 Unclassified_Cell_type_specific 	   2.859016  1.32460816
## 		      t      P.Value   adj.P.Val	B
## cg07499259  18.73131 7.267204e-08 0.005067836 7.453206
## cg26992245  18.32674 8.615461e-08 0.005067836 7.359096
## cg09747445 -18.24438 8.923101e-08 0.005067836 7.339443
## cg18808929 -17.90181 1.034217e-07 0.005067836 7.255825
## cg25015733 -17.32615 1.333546e-07 0.005067836 7.108231
## cg21179654  17.27804 1.362674e-07 0.005067836 7.095476
                    


The resulting data.frame can easily be written to a CSV file, which can be opened in Excel.



                        write.table
                        (DMPs, 
                        file=
                        "DMPs.csv"
                        , 
                        sep=
                        ","
                        , 
                        row.names=
                        FALSE
                        )
                    


**Figure 9.**
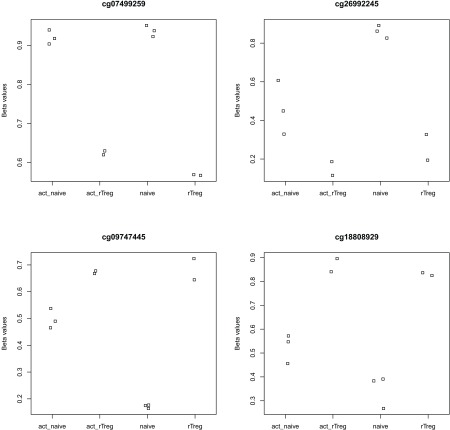
Plotting the top few differentially methylated CpGs is a good way to check whether the results make sense.



It is always useful to plot sample-wise methylation levels for the top differentially methylated CpG sites to quickly ensure the results make sense ( Figure 9). If the plots do not look as expected, it is usually an indication of an error in the code, or in setting up the design matrix. It is easier to interpret methylation levels on the beta value scale, so although the analysis is performed on the M-value scale, we visualise data on the beta value scale. The plotCpg function in *minfi* is a convenient way to plot the sample-wise beta values stratified by the grouping variable.



                        # plot the top 4 most significantly differentially methylated CpGs

                        par
                        (
                        mfrow=c
                        (
                        2
                        ,
                        2
                        ))

                        sapply
                        (
                        rownames
                        (DMPs)[
                        1
                        :
                        4
                        ], 
                        function(cpg){
  
                        plotCpg
                        (
                        bVals, 
                        cpg=
                        cpg, 
                        pheno=
                        targets$Sample_Group, 
                        ylab = 
                        "Beta values"
                        )
})
                    




                        ## $cg07499259
## NULL
##
## $cg26992245
## NULL
##
## $cg09747445
## NULL
##
## $cg18808929
## NULL
                    


### Differential methylation analysis of regions

Although performing a *probe-wise* analysis is useful and informative, sometimes we are interested in knowing whether several proximal CpGs are concordantly differentially methylated, that is, we want to identify differentially methylated *regions*. There are several Bioconductor packages that have functions for identifying differentially methylated regions from 450k data. Some of the most popular are the dmrFind function in the charm package, which has been somewhat superseded for 450k arrays by the bumphunter function in minfi( Aryee *et al.*, 2014; Jaffe *et al.*, 2012), and, the recently published dmrcate in the DMRcate package ( Peters *et al.*, 2015). They are each based on different statistical methods. In our experience, the bumphunter and dmrFind functions can be somewhat slow to run unless you have the computer infrastructure to parallelise them, as they use permutations to assign significance. In this workflow, we will perform an analysis using the dmrcate. As it is based on *limma*, we can directly use the design and contMatrix we previously defined.

Firstly, our matrix of M-values is annotated with the relevant information about the probes such as their genomic position, gene annotation, etc. By default, this is done using the ilmn12.hg19 annotation, but this can be substituted for any argument compatible with the interface provided by the *minfi* package. The *limma* pipeline is then used for differential methylation analysis to calculate moderated t-statistics.

                        myAnnotation <- 
                        cpg.annotate
                        (
                        object = 
                        mVals, 
                        datatype = 
                        "array"
                        , 
                        what = 
                        "M"
                        ,
                                
                        analysis.type= 
                        "differential"
                        , 
                        design = 
                        design,
                                
                        contrasts = 
                        TRUE
                        , 
                        cont.matrix = 
                        contMatrix,
                                
                        coef = 
                        "naive - rTreg"
                        , 
                        arraytype = 
                        "450K"
                        )
                    




 
                        ## Your contrast returned 3021 individually significant probes.
 
                        ## We recommend the default setting of pcutoff in dmrcate().
                    




                        str
                        (myAnnotation)
                    




                        ## List of 7
## $ ID   :Factor w/ 439918 levels "cg00000029","cg00000108",..: 232388 391918 260351 ...
##  $ stat  : num [1:439918] 0.0489 -2.0773 0.7711 -0.0304 -0.764 ...
##  $ CHR   : Factor w/ 24 levels "chr1","chr10",..: 1 1 1 1 1 1 1 1 1 1 ...
##  $ pos   : int [1:439918] 15865 534242 710097 714177 720865 758829 763119 779995 ...
##  $ betafc: num [1:439918] 0.00039 -0.04534 0.01594 0.00251 -0.00869 ...
##  $ indfdr: num [1:439918] 0.994 0.565 0.872 0.997 0.873 ...
##  $ is.sig: logi [1:439918] FALSE FALSE FALSE FALSE FALSE FALSE ...
##  - attr(*, "row.names")= int [1:439918] 1 2 3 4 5 6 7 8 9 10 ...
##  - attr(*, "class")= chr "annot"
                    


Once we have the relevant statistics for the individual CpGs, we can then use the dmrcate function to combine them to identify differentially methylated regions. The main output table DMRs$results contains all of the regions found, along with their genomic annotations and p-values.



                        DMRs <- 
                        dmrcate
                        (myAnnotation, 
                        lambda=
                        1000
                        , 
                        C=
                        2
                        )

                        head
                        (DMRs$results)
                    




                        ##                          coord no.cpgs        minfdr     Stouffer
## 452    chr17:57915665-57918682      12  4.957890e-91 6.639928e-10
## 723   chr3:114012316-114012912       5 1.622885e-180 1.515378e-07
## 464    chr17:74639731-74640078       6  9.516873e-90 1.527961e-07
## 1053    chrX:49121205-49122718       6  6.753751e-84 2.936984e-07
## 487    chr18:21452730-21453131       7 5.702319e-115 7.674943e-07
## 186  chr10:135202522-135203200       6  1.465070e-65 7.918224e-07
##       maxbetafc meanbetafc
## 452   0.3982862  0.3131611
## 723   0.5434277  0.4251622
## 464  -0.2528645 -0.1951904
## 1053  0.4529088  0.3006242
## 487  -0.3867474 -0.2546089
## 186   0.2803157  0.2293419
                    


**Figure 10.**
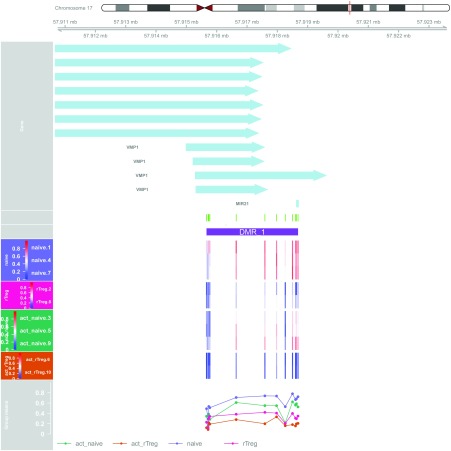
The DMRcate “DMR.plot” function allows you to quickly visualise DMRs in their genomic context. By default, the plot shows the location of the DMR in the genome, the position of any genes that are nearby, the base pair positions of the CpG probes, the methylation levels of the individual samples as a heatmap and the mean methylation levels for the various sample groups in the experiment. This plot shows the top ranked DMR identified by the DMRcate analysis.

As for the probe-wise analysis, it is advisable to visualise the results to ensure that they make sense. The regions can easily be viewed using the DMR.plot function provided in the *DMRcate* package ( Figure 10).



                        # convert the regions to annotated genomic ranges

                        data
                        (dmrcatedata)
results.ranges <- 
                        extractRanges
                        (DMRs, 
                        genome = 
                        "hg19"
                        )
                    




                        # set up the grouping variables and colours

                        groups <- pal[
                        1
                        :
                        length
                        (
                        unique
                        (targets$Sample_Group))]

                        names
                        (groups) <- 
                        levels
                        (
                        factor
                        (targets$Sample_Group))
cols <- groups[
                        as.character
                        (
                        factor
                        (targets$Sample_Group))]
samps <- 
                        1
                        :
                        nrow
                        (targets)
                    




                        # draw the plot for the top DMR

                        par
                        (
                        mfrow=c
                        (
                        1
                        ,
                        1
                        ))

                        DMR.plot(ranges=
                        results.ranges, 
                        dmr=
                        1
                        , 
                        CpGs=
                        bVals, 
                        phen.col=
                        cols,
                         what = 
                        "Beta"
                        ,
          
                        arraytype = 
                        "450K"
                        , 
                        pch=
                        16
                        , 
                        toscale=
                        TRUE
                        , 
                        plotmedians=
                        TRUE
                        , 
          
                        genome=
                        "hg19"
                        , 
                        samps=
                        samps)
                    


### Customising visualisations of methylation data

**Figure 11.**
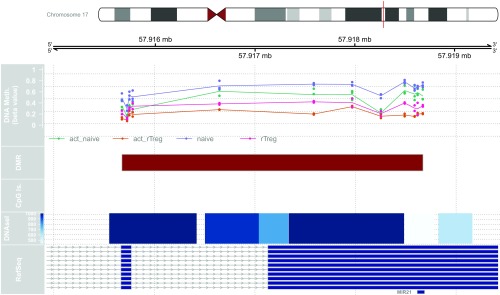
The Gviz package provides extensive functionality for customising plots of genomic regions. This plot shows the top ranked DMR identified by the DMRcate analysis.

The *Gviz* package offers powerful functionality for plotting methylation data in its genomic context. The package vignette is very extensive and covers the various types of plots that can be produced using the *Gviz* framework. We will plot one of the differentially methylated regions from the *DMRcate* analysis to demonstrate the type of visualisations that can be created ( Figure 11).

We will first set up the genomic region we would like to plot by extracting the genomic coordinates of the top differentially methylated region.



                        # indicate which genome is being used

                        gen <- 
                        "hg19"

                        # the index of the DMR that we will plot

                        dmrIndex <- 
                        1

                        # extract chromosome number and location from DMR results

                        coords <- 
                        strsplit2
                        (DMRs$results$coord[dmrIndex],
                        ":"
                        )
chrom <- coords[
                        1
                        ]
start <- 
                        as.numeric(
                        strsplit2
                        (coords[
                        2
                        ],
                        "-"
                        )[
                        1
                        ])
end <- 
                        as.numeric
                        (
                        strsplit2
                        (coords[
                        2
                        ],
                        "-"
                        )[
                        2])

                        # add 25% extra space to plot

                        minbase <- start - (
                        0.25
                        *(end-start))
maxbase <- end + (
                        0.25
                        *(end-start))
                    


Next, we will add some genomic annotations of interest such as the locations of CpG islands and DNAseI hypersensitive sites; this can be any feature or genomic annotation of interest that you have data available for. The CpG islands data was generated using the method published by Wu *et al.* (2010); the DNaseI hypersensitive site data was obtained from the UCSC Genome Browser.



                        # CpG islands

                        islandHMM <- 
                        read.csv
                        (
                        paste
                        (dataDirectory,
                               
                        "model-based-cpg-islands-hg19-chr17.txt"
                        , 
                        sep=
                        "/"
                        ), 
                        
                        sep=
                        "\t"
                        , 
                        stringsAsFactors=
                        FALSE
                        , 
                        header=
                        FALSE
                        )

                        head
                        (islandHMM)
                    




                        ##                V1      V2     V3    V4   V5   V6    V7    V8
## 1 chr17_ctg5_hap1    8935  10075  1141  129  815 0.714 0.887
## 2 chr17_ctg5_hap1   64252  64478   227   30  165 0.727 1.014
## 3 chr17_ctg5_hap1   87730  89480  1751  135 1194 0.682 0.663
## 4 chr17_ctg5_hap1   98265  98591   327   29  226 0.691 0.744
## 5 chr17_ctg5_hap1  120763 125451  4689  359 3032 0.647 0.733
## 6 chr17_ctg5_hap1  146257 146607   351   19  231 0.658 0.500
                    




                        islandData <- 
                        GRanges
                        (
                        seqnames=Rle
                        (islandHMM[,
                        1
                        ]),
                        
                        ranges=IRanges
                        (
                        start=
                        islandHMM[,
                        2
                        ], 
                        end=
                        islandHMM[,
                        3
                        ]),
                        
                        strand=Rle
                        (
                        strand
                        (
                        rep
                        (
                        "*"
                        ,
                        nrow
                        (islandHMM)))))
islandData
                    




                        ## GRanges    object with 3456 ranges and 0 metadata columns:
##                    seqnames               ranges strand
##                       <Rle>            <IRanges>  <Rle>
##      [1]    chr17_ctg5_hap1     [   8935, 10075]      *
##      [2]    chr17_ctg5_hap1     [  64252, 64478]      *
##      [3]    chr17_ctg5_hap1     [  87730, 89480]      *
##      [4]    chr17_ctg5_hap1     [  98265, 98591]      *
##      [5]    chr17_ctg5_hap1     [120763, 125451]      *
##      ...      ...                   ...   ...
##   [3452]              chr17 [81147380, 81147511]      *
##   [3453]              chr17 [81147844, 81148321]      *
##   [3454]              chr17 [81152612, 81153665]      *
##   [3455]              chr17 [81156194, 81156512]      *
##   [3456]              chr17 [81162945, 81165532]      *
##   -------
##   seqinfo: 5 sequence from an unspecified genome; no seqlengths
                    




                        # DNAseI hypersensitive sites

                        dnase <- 
                        read.csv
                        (
                        paste
                        (dataDirectory,
                        "wgEncodeRegDnaseClusteredV3chr17.bed"
                        ,
                           
                        sep=
                        "/"
                        ),
                    
                        sep=
                        "\t"
                        ,
                        stringsAsFactors=
                        FALSE
                        ,
                        header=
                        FALSE
                        )

                        head
                        (dnase)
                    




                        ##      V1   V2   V3 V4  V5 V6                                       V7
## 1 chr17  125  335  7 444  7                    84,83,88,90,77,87,89,
## 2 chr17  685  835  1 150  1                                      80,
## 3 chr17 2440 2675 13 410 13 0,30,102,104,38,47,61,68,122,1,51,73,75,
## 4 chr17 3020 3170  1 247  1                                     120,
## 5 chr17 3740 3890  2 161  2                                   71,73,
## 6 chr17 5520 6110  4 241  5                          17,19,25,16,16,
##                                               V8
## 1                   328,208,444,218,109,171,191,
## 2                                           150, 
## 3 204,410,301,206,46,48,84,164,85,12,98,215,146,
## 4                                           247,
## 5                                       108,161,
## 6                             241,185,239,26,52,
dnaseData <- 
                        GRanges
                        (
                        seqnames=
                        dnase[,
                        1
                        ],
		        
                        ranges=IRanges
                        (
                        start=
                        dnase[,
                        2
                        ], 
                        end=
                        dnase[,
                        3
                        ]),
                        
                        strand=Rle
                        (
                        rep
                        (
                        "*"
                        ,
                        nrow
                        (dnase))),
                        
                        data=
                        dnase[,
                        5])

                        dnaseData

##  GRanges  object with 74282 ranges and 1 metadata column:
##	       seqnames	              ranges strand |	   data
##		  <Rle>	           <IRanges>  <Rle> | <integer>
##	   [1]	  chr17         [  125, 335]	  * |	    444
##	   [2]	  chr17         [  685, 835]	  * |	    150
##	   [3]	  chr17         [2440, 2675]	  * |	    410
##	   [4]	  chr17         [3020, 3170]	  * |	    247
##	   [5]	  chr17         [3740, 3890]	  * |	    161
##	   ...	    ...	                 ...    ... .	    ...
##     [74278]	  chr17 [81153140, 81153350]	  * |	    574
##     [74279]	  chr17 [81153580, 81153810]	  * |	    208
##     [74280]	  chr17 [81185540, 81185750]	  * |	    326
##     [74281]	  chr17 [81188880, 81189090]	  * |	    209
##     [74282]	  chr17 [81194900, 81195115]	  * |	    185
##     -------
##     seqinfo: 1 sequence from an unspecified genome; no seqlengths

                    


Now, set up the ideogram, genome and RefSeq tracks that will provide context for our methylation data.



                        iTrack <- 
                        IdeogramTrack
                        (
                        genome = 
                        gen, 
                        chromosome = 
                        chrom, 
                        name=
                        ""
                        )
gTrack <- 
                        GenomeAxisTrack
                        (
                        col=
                        "black"
                        , 
                        cex=
                        1
                        , 
                        name=
                        ""
                        , 
                        fontcolor=
                        "black"
                        )
rTrack <- 
                        UcscTrack
                        (
                        genome=
                        gen, 
                        chromosome=
                        chrom, 
                        track=
                        "refGene"
                        ,
                        
                        from=
                        minbase, 
                        to=
                        maxbase, 
                        trackType=
                        "GeneRegionTrack"
                        ,
                        
                        rstarts=
                        "exonStarts"
                        , 
                        rends=
                        "exonEnds"
                        , 
                        gene=
                        "name"
                        ,
                        
                        symbol=
                        "name2"
                        , 
                        transcript=
                        "name"
                        , 
                        strand=
                        "strand"
                        ,
                        
                        fill=
                        "darkblue"
                        ,
                        stacking=
                        "squish"
                        , 
                        name=
                        "RefSeq"
                        ,
                        
                        showId=
                        TRUE
                        , 
                        geneSymbol=
                        TRUE
                        )



Ensure that the methylation data is ordered by chromosome and base position.



                        ann450kOrd <- ann450kSub[
                        order
                        (ann450kSub$chr,ann450kSub$pos),]

                        head
                        (ann450kOrd)


## DataFrame with 6 rows and 22 columns
##	              chr	pos	 strand	       Name	Probe_rs
##	      <character> <integer> <character> <character>  <character>
## cg13869341	     chr1     15865	      +	 cg13869341	      NA
## cg24669183	     chr1    534242	      -	 cg24669183    rs6680725
## cg15560884	     chr1    710097           +	 cg15560884	      NA
## cg01014490        chr1    714177	      -	 cg01014490	      NA
## cg17505339	     chr1    720865	      -	 cg17505339	      NA
## cg11954957	     chr1    758829	      +	 cg11954957  rs115498424
##	      Probe_maf      CpG_rs   CpG_maf      SBE_rs     SBE_maf
##	      <numeric> <character> <numeric> <character>   <numeric>
## cg13869341	     NA	         NA	   NA	       NA	   NA
## cg24669183  0.108100	         NA	   NA	       NA	   NA
## cg15560884	     NA	         NA	   NA	       NA	   NA
## cg01014490	     NA	         NA	   NA	       NA	   NA
## cg17505339	     NA	         NA	   NA	       NA	   NA
## cg11954957  0.029514	         NA	   NA	       NA	   NA
##	            Islands_Name Relation_to_Island UCSC_RefGene_Name
##                   <character>	<character>	  <character>
## cg13869341	                            OpenSea	       WASH5P
## cg24669183  chr1:533219-534114	    S_Shore
## cg15560884  chr1:713984-714547	    N_Shelf
## cg01014490  chr1:713984-714547	     Island
## cg17505339	                            OpenSea
## cg11954957  chr1:762416-763445	    N_Shelf
##	       UCSC_RefGene_Accession UCSC_RefGene_Group     Phantom
##	                  <character>	     <character> <character>
## cg13869341	            NR_024540	            Body
## cg24669183
## cg15560884
## cg01014490
## cg17505339
## cg11954957 
##	              DMR      Enhancer      HMM_Island Regulatory_Feature_Name
##	      <character>   <character>	    <character>	            <character>
## cg13869341
## cg24669183	                        1:523025-524193
## cg15560884
## cg01014490	                        1:703784-704410	        1:713802-715219
## cg17505339
## cg11954957
##	      Regulatory_Feature_Group	       DHS
##	                   <character> <character>
## cg13869341
## cg24669183
## cg15560884
## cg01014490	  Promoter_Associated
## cg17505339
## cg11954957


bValsOrd <- bVals[
                        match
                        (ann450kOrd$Name,
                        rownames
                        (bVals)),]

                        head
                        (bValsOrd)


##               naive.1    rTreg.2 act_naive.3    naive.4 act_naive.5
## cg13869341 0.84267937 0.85118462   0.8177504 0.82987650  0.81186174
## cg24669183 0.81812908 0.82489238   0.8293297 0.75610281  0.81967323
## cg15560884 0.77219626 0.74903910   0.7516263 0.77417882  0.77266205
## cg01014490 0.08098986 0.06590459   0.0233755 0.04127262  0.04842397
## cg17505339 0.89439216 0.93822870   0.9471357 0.90520570  0.92641305
## cg11954957 0.74495496 0.79008516   0.7681146 0.84450764  0.75431167
##            act_rTreg.6   naive.7    rTreg.8 act_naive.9 act_rTreg.10
## cg13869341   0.8090798 0.8891851 0.88537940  0.90916748   0.88334231
## cg24669183   0.8187838 0.7903763 0.85304116  0.80930568   0.80979554
## cg15560884   0.7721528 0.7658623 0.75909061  0.78099397   0.78569274
## cg01014490   0.0644404 0.0245281 0.02832358  0.07740468   0.04640659
## cg17505339   0.9286016 0.8889361 0.87205348  0.90099782   0.93508348
## cg11954957   0.8116911 0.7832207 0.84929777  0.84719430   0.83350220




Create the data tracks using the appropriate track type for each data type.




                        # create genomic ranges object from methylation data

                        cpgData <- 
                        GRanges
                        (
                        seqnames=Rle
                        (ann450kOrd$chr),
                     
                        ranges=IRanges
                        (
                        start=
                        ann450kOrd$pos, 
                        end=
                        ann450kOrd$pos),
                     
                        strand=Rle
                        (
                        rep
                        (
                        "*"
                        ,
                        nrow
                        (ann450kOrd))),
                     
                        betas=
                        bValsOrd)

                        # extract data on CpGs in DMR

                        cpgData <- 
                        subsetByOverlaps
                        (cpgData, results.ranges[dmrIndex])


                        # methylation data track

                        methTrack <- 
                        DataTrack
                        (
                        range=
                        cpgData, 
                        groups=
                        targets$Sample_Group,
                        genome = 
                        gen,
                          
                        chromosome=
                        chrom, 
                        ylim=c
                        (-
                        0.05
                        ,
                        1.05
                        ), 
                        col=
                        pal,
                          
                        type=c
                        (
                        "a"
                        ,
                        "p"
                        ), 
                        name=
                        "DNA Meth.\n(beta value)"
                        ,
                          
                        background.panel=
                        "white"
                        , 
                        legend=
                        TRUE
                        , 
                        cex.title=
                        0.8
                        ,
                          
                        cex.axis=
                        0.8
                        , 
                        cex.legend=
                        0.8
                        )

                        # CpG island track

                        islandTrack <- 
                        AnnotationTrack
                        (
                        range=
                        islandData, 
                        genome=
                        gen, 
                        name=
                        "CpG Is."
                        ,
                                   
                        chromosome=
                        chrom,
                        fill=
                        "darkgreen"
                        )


                        # DNaseI hypersensitive site data track

                        dnaseTrack <- 
                        DataTrack
                        (
                        range=
                        dnaseData, 
                        genome=
                        gen, 
                        name=
                        "DNAseI"
                        ,
                           
                        type=
                        "gradient"
                        , 
                        chromosome=
                        chrom)


                        # DMR position data track

                        dmrTrack <- 
                        AnnotationTrack
                        (
                        start=
                        start, 
                        end=
                        end, 
                        genome=
                        gen,  
                        name=
                        "DMR"
                        ,
                               
                        chromosome=
                        chrom,
                        fill=
                        "darkred"
                        )
                    


Set up the track list and indicate the relative sizes of the different tracks. Finally, draw the plot using the plotTracks function ( Figure 11).



                        tracks <-  
                        list
                        (iTrack, gTrack, methTrack, dmrTrack, islandTrack, dnaseTrack,
		
                            rTrack)
sizes <- 
                        c
                        (
                        2
                        ,
                        2
                        ,
                        5
                        ,
                        2
                        ,
                        2
                        ,
                        2
                        ,
                        3
                        )  
                        # set up the relative sizes of the tracks

                        plotTracks
                        (tracks, 
                        from=
                        minbase, 
                        to=
                        maxbase, 
                        showTitle=
                        TRUE
                        , 
                        add53=
                        TRUE
                        ,
            
                        add35=
                        TRUE
                        , 
                        grid=
                        TRUE
                        , 
                        lty.grid=
                        3
                        , 
                        sizes=
                        sizes, 
                        length
                        (tracks))
                    


## Additional analyses

### Gene ontology testing

Once you have performed a differential methylation analysis, there may be a very long list of significant CpG sites to interpret. One question a researcher may have is, “which gene pathways are over-represented for differentially methylated CpGs?” In some cases it is relatively straightforward to link the top differentially methylated CpGs to genes that make biological sense in terms of the cell types or samples being studied, but there may be many thousands of CpGs significantly differentially methylated. In order to gain an understanding of the biological processes that the differentially methylated CpGs may be involved in, we can perform gene ontology or KEGG pathway analysis using the gometh function in the *missMethyl* package ( Phipson *et al.*, 2016).

Let us consider the first comparison, naive vs rTreg, with the results of the analysis in the DMPs table. The gometh function takes as input a character vector of the names (e.g. cg20832020) of the significant CpG sites, and optionally, a character vector of all CpGs tested. This is recommended particularly if extensive filtering of the CpGs has been performed prior to analysis. For gene ontology testing (default), the user can specify collection="GO”. For testing KEGG pathways, specify collection="KEGG”. In the DMPs table, the Name column corresponds to the CpG name. We will select all CpG sites that have adjusted p-value of less than 0.05.



                        # Get the significant CpG sites at less than 5% FDR

                        sigCpGs <- DMPs$Name[DMPs$adj.P.Val<
                        0.05
                        ]

                        # First 10 significant CpGs

                        sigCpGs[
                        1
                        :
                        10
                        ]


##  [1] "cg07499259" "cg26992245" "cg09747445" "cg18808929" "cg25015733"
##  [6] "cg21179654" "cg26280976" "cg16943019" "cg10898310" "cg25130381"



                        # Total number of significant CpGs at 5% FDR

                        length
                        (sigCpGs)


                        ## [1] 3021


                        # Get all the CpG sites used in the analysis to form the background

                        all <- DMPs$Name

                        # Total number of CpG sites tested

                        length
                        (all)


## [1] 439918



**Figure 12.**
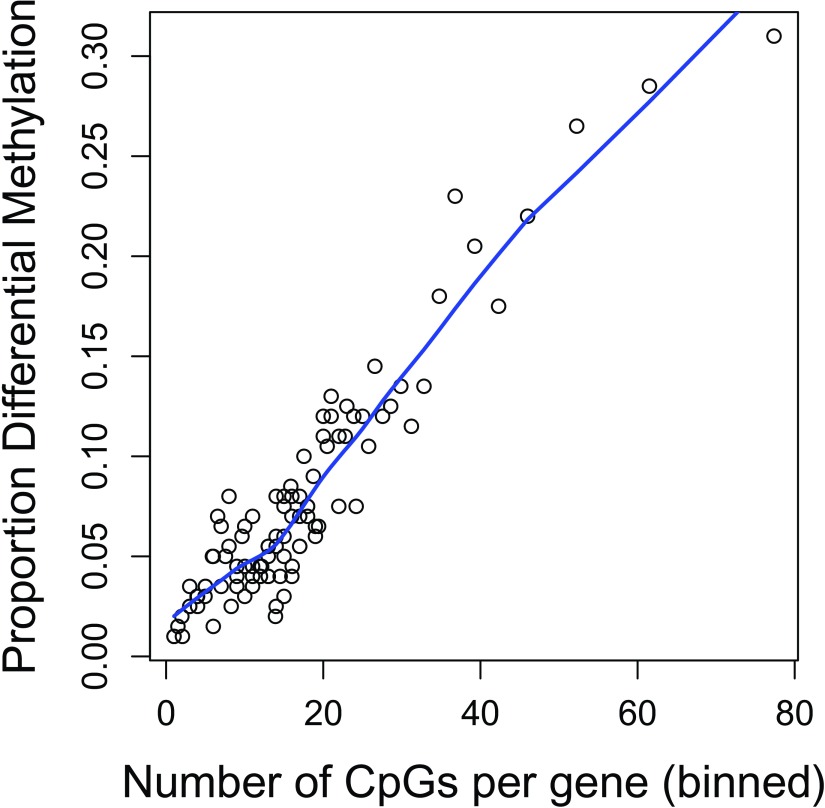
Bias resulting from different numbers of CpG probes in different genes.



The gometh function takes into account the varying numbers of CpGs associated with each gene on the Illumina methylation arrays. For the 450k array, the numbers of CpGs mapping to genes can vary from as few as 1 to as many as 1200. The genes that have more CpGs associated with them will have a higher probability of being identified as differentially methylated compared to genes with fewer CpGs. We can look at this bias in the data by specifying plot=TRUE in the call to gometh ( Figure 12).

                        par
                        (
                        mfrow=c
                        (
                        1
                        ,
                        1
                        ))
gst <- 
                        gometh
                        (
                        sig.cpg=
                        sigCpGs, 
                        all.cpg=
                        all, 
                        plot.bias=
                        TRUE
                        )



                        ## Warning in alias2SymbolTable(flat$symbol): Multiple symbols ignored for one
## or more aliases
                    


The gst object is a data.frame with each row corresponding to the GO category being tested. Note that the warning regarding multiple symbols will always be displayed as there are genes that have more than one alias, however it is not a cause for concern.

The top 20 gene ontology categories can be displayed using the topGO function. For KEGG pathway analysis, the topKEGG function can be called to display the top 20 enriched pathways.




                        # Top 10 GO categories

                        topGO
                        (gst, 
                        number=
                        10
                        )


##	                                     Term Ont    N  DE
## GO:0002376	            immune system process  BP 2240 340
## GO:0006955	                  immune response  BP 1409 212
## GO:0001775	                  cell activation  BP  837 158
## GO:0007159	     leukocyte cell-cell adhesion  BP  455 103
## GO:0046649	            lymphocyte activation  BP  574 119
## GO:0002682 regulation of immune system process  BP 1225 195
## GO:0045321	             leukocyte activation  BP  676 130
## GO:0070486	            leukocyte aggregation  BP  423  96
## GO:0042110	                T cell activation  BP  415  94 
## GO:0070489	               T cell aggregation  BP  415  94
##	                                        P.DE
## GO:0002376 0.000000000000000000000000000003229702
## GO:0006955 0.000000000000000000000422272703517178
## GO:0001775 0.000000000000000000010295538258512461
## GO:0007159 0.000000000000000000090040070213398411
## GO:0046649 0.000000000000000000250620553154991038
## GO:0002682 0.000000000000000000263741544330346864
## GO:0045321 0.000000000000000001995676602987099282
## GO:0070486 0.000000000000000002407114373683902864
## GO:0042110 0.000000000000000004264084670812330066
## GO:0070489 0.000000000000000004264084670812330066
##                                           FDR
## GO:0002376 0.00000000000000000000000006820484
## GO:0006955 0.00000000000000000445877747643788
## GO:0001775 0.00000000000000007247372564775539
## GO:0007159 0.00000000000000047536655069163696
## GO:0046649 0.00000000000000092828232219471078
## GO:0002682 0.00000000000000092828232219471078
## GO:0045321 0.00000000000000602067121455450880
## GO:0070486 0.00000000000000635418016793208260
## GO:0042110 0.00000000000000900489400782147891
## GO:0070489 0.00000000000000900489400782147891
                    


From the output we can see many of the top GO categories correspond to immune system and T cell processes, which is unsurprising as the cell types being studied form part of the immune system. Typically, we consider GO categories that have associated false discovery rates of less than 5% to be statistically significant. If there aren’t any categories that achieve this significance it may be useful to scan the top 5 or 10 highly ranked GO categories to gain some insight into the biological system.

The gometh function only tests GO and KEGG pathways. For a more generalised version of gene set testing for methylation data where the user can specify the gene set to be tested, the gsameth function can be used. To display the top 20 pathways, topGSA can be called. gsameth accepts a single gene set, or a list of gene sets. The gene identifiers in the gene set must be Entrez Gene IDs. To demonstrate gsameth, we are using the curated genesets (C2) from the Broad Institute Molecular signatures database. These can be downloaded as an RData object from the WEHI Bioinformatics website.



                        # load Broad human curated (C2) gene sets

                        load
                        (
                        paste
                        (dataDirectory,
                        "human_c2_v5.rdata"
                        ,
                        sep=
                        "/"
                        ))

                        # perform the gene set test(s)

                        gsa <- 
                        gsameth
                        (
                        sig.cpg=
                        sigCpGs, 
                        all.cpg=
                        all, 
                        collection=
                        Hs.c2)





                        ## Warning in alias2SymbolTable(flat$symbol): Multiple symbols ignored for one
## or more aliases
                    




                        # top 10 gene sets

                        topGSA
                        (gsa, 
                        number=
                        10
                        )
                    




                        ##                                         N  DE                       P.DE
## REACTOME_IMMUNE_SYSTEM                933 127 0.000000000000000000000000
## DACOSTA_UV_RESPONSE_VIA_ERCC3_DN      855 147 0.000000000000000000000000
## ZHENG_BOUND_BY_FOXP3                  491 138 0.000000000000000000000000
## MARSON_BOUND_BY_FOXP3_UNSTIMULATED   1229 169 0.000000000000000000000000
## CHEN_METABOLIC_SYNDROM_NETWORK       1210 162 0.000000000000000000000000
## MARTENS_BOUND_BY_PML_RARA_FUSION      456 105 0.000000000000000000000000
## PILON_KLF1_TARGETS_DN                1972 262 0.000000000000000000000000
## JAATINEN_HEMATOPOIETIC_STEM_CELL_DN   226  59 0.000000000000000004151028
## SMID_BREAST_CANCER_NORMAL_LIKE_UP     476  92 0.000000000000002674784772
## LEE_EARLY_T_LYMPHOCYTE_DN              57  25 0.000000000000825660086765
##                                                          FDR
## REACTOME_IMMUNE_SYSTEM               0.000000000000000000000
## DACOSTA_UV_RESPONSE_VIA_ERCC3_DN     0.000000000000000000000
## ZHENG_BOUND_BY_FOXP3                 0.000000000000000000000
## MARSON_BOUND_BY_FOXP3_UNSTIMULATED   0.000000000000000000000
## CHEN_METABOLIC_SYNDROM_NETWORK       0.000000000000000000000
## MARTENS_BOUND_BY_PML_RARA_FUSION     0.000000000000000000000
## PILON_KLF1_TARGETS_DN                0.000000000000000000000
## JAATINEN_HEMATOPOIETIC_STEM_CELL_DN  0.000000000000002451701
## SMID_BREAST_CANCER_NORMAL_LIKE_UP    0.000000000001404262005
## LEE_EARLY_T_LYMPHOCYTE_DN            0.000000000390124390997
                    


While gene set testing is useful for providing some biological insight in terms of what pathways might be affected by abberant methylation, care should be taken not to over-interpret the results. Gene set testing should be used for the purpose of providing some biological insight that ideally would be tested and validated in further laboratory experiments. It is important to keep in mind that we are not observing gene level activity such as in RNA-Seq experiments, and that we have had to take an extra step to associate CpGs with genes.

### Differential variability

Rather than testing for differences in mean methylation, we may be interested in testing for differences between group variances. For example, it has been hypothesised that highly variable CpGs in cancer may contribute to tumour heterogeneity ( Hansen *et al.*, 2011). Hence we may be interested in CpG sites that are consistently methylated in one group, but variably methylated in another group.

Sample size is an important consideration when testing for differentially variable CpG sites. In order to get an accurate estimate of the group variances, larger sample sizes are required than for estimating group means. A good rule of thumb is to have at least ten samples in each group ( Phipson & Oshlack, 2014). To demonstrate testing for differentially variable CpG sites, we will use a publicly available dataset on ageing GSE30870, where whole blood samples were collected from 18 centenarians and 18 newborns and profiled for methylation on the 450k array ( Heyn *et al.*, 2012). The data ( age.rgSet) and sample information ( age.targets) have been included as an R data object in the data archive you previously downloaded from figshare. We can load the data using the load command, after which it needs to be normalised and filtered as previously described.



                        # load data

                        load
                        (
                        paste
                        (dataDirectory,
                        "ageData.RData"
                        ,
                        sep=
                        "/"
                        ))


                        # calculate detection p-values

                        age.detP <- 
                        detectionP
                        (age.rgSet)


                        # pre-process the data after excluding poor quality samples

                        age.mSetSq <- 
                        preprocessQuantile
                        (age.rgSet)
                    




                        ## [preprocessQuantile] Mapping to genome.
                    




                        ## [preprocessQuantile] Fixing outliers.
                    




                        ## [preprocessQuantile] Quantile normalizing.
                    




                        # add sex information to targets information

                        age.targets$Sex <- 
                        getSex
                        (age.mSetSq)$predictedSex


                        # ensure probes are in the same order in the mSetSq and detP objects

                        age.detP <- age.detP[
                        match
                        (
                        featureNames
                        (age.mSetSq),
                        rownames
                        (age.detP)),]

                        # remove poor quality probes

                        keep <- 
                        rowSums
                        (age.detP < 
                        0.01
                        ) == 
                        ncol
                        (age.detP)
age.mSetSqFlt <- age.mSetSq[keep,]


                        # remove probes with SNPs at CpG or single base extension (SBE) site

                        age.mSetSqFlt <- 
                        dropLociWithSnps
                        (age.mSetSqFlt, 
                        snps = c
                        (
                        "CpG"
                        , 
                        "SBE"
                        ))


                        # remove cross-reactive probes

                        keep <- !(
                        featureNames
                        (age.mSetSqFlt) %in% xReactiveProbes$TargetID)
age.mSetSqFlt <- age.mSetSqFlt[keep,]
                    


**Figure 13.**
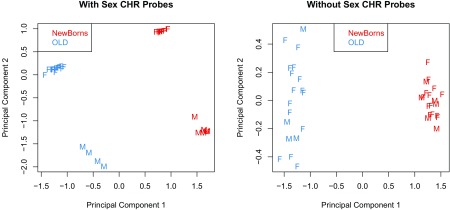
When samples from both males and females are included in a study, sex is usually the largest source of variation in methylation data.

As this dataset contains samples from both males and females, we can use it to demonstrate the effect of removing sex chromosome probes on the data. The MDS plots below show the relationship between the samples in the ageing dataset before and after sex chromosome probe removal ( Figure 13). It is apparent that before the removal of sex chromosome probes, the sample cluster based on sex in the second principal component. When the sex chromosome probes are removed, age is the largest source of variation present and the male and female samples no longer form separate clusters.



                        # tag sex chromosome probes for removal

                        keep <- !(
                        featureNames
                        (age.mSetSqFlt) %in% ann450k$Name[ann450k$chr %in%
								   
                         c
                        (
                        "chrX"
                        ,
                        "chrY"
                        )])
age.pal <- 
                        brewer.pal
                        (
                        8
                        ,
                        "Set1"
                        )

                        par
                        (
                        mfrow=c
                        (
                        1
                        ,
                        2
                        ))

                        plotMDS
                        (
                        getM
                        (age.mSetSqFlt), 
                        top=
                        1000
                        , 
                        gene.selection=
                        "common"
                        ,
	 
                        col=
                        age.pal[
                        factor
                        (age.targets$Sample_Group)], 
                        labels=
                        age.targets$Sex,
	 
                        main=
                        "With Sex CHR Probes"
                        )

                        legend
                        (
                        "topleft"
                        , 
                        legend=levels
                        (
                        factor
                        (age.targets$Sample_Group)),
	
                        text.col=
                        age.pal)


                        plotMDS
                        (
                        getM
                        (age.mSetSqFlt[keep,]), 
                        top=
                        1000
                        , 
                        gene.selection=
                        "common"
                        ,
	 
                        col=
                        age.pal[
                        factor
                        (age.targets$Sample_Group)], 
                        labels=
                        age.targets$Sex,
	 
                        main=
                        "Without Sex CHR Probes"
                        )

                        legend
                        (
                        "top"
                        , 
                        legend=levels
                        (
                        factor
                        (age.targets$Sample_Group)),
	
                        text.col=
                        age.pal)
                    




                        # remove sex chromosome probes from data

                        age.mSetSqFlt <- age.mSetSqFlt[keep,]
                    


We can test for differentially variable CpGs using the varFit function in the *missMethyl* package. The syntax for specifying which groups we are interested in testing is slightly different to the standard way a model is specified in limma, particularly for designs where an intercept is fitted (see *missMethyl* vignette for further details). For the ageing data, the design matrix includes an intercept term, and a term for age. The coef argument in the varFit function indicates which columns of the design matrix correspond to the intercept and grouping factor. Thus, for the ageing dataset we set coef=c(1,2). Note that design matrices without intercept terms are permitted, with specific contrasts tested using the contrasts.varFit function.



                        # get M-values for analysis

                        age.mVals <- 
                        getM
                        (age.mSetSqFlt)

design <- 
                        model.matrix
                        (~
                        factor
                        (age.targets$Sample_Group))

                        # Fit the model for differential variability
# specifying the intercept and age as the grouping factor

                        fitvar <- 
                        varFit
                        (age.mVals, 
                        design = 
                        design, 
                        coef = c
                        (
                        1
                        ,
                        2
                        ))


                        # Summary of differential variability

                        summary
                        (
                        decideTests
                        (fitvar))
                    




                        ##    (Intercept) factor(age.targets$Sample_Group)OLD
## -1 		0 				 1325
## 0 	    11441 			       393451
## 1 	   417787 				34452
                    




                        topDV <- 
                        topVar
                        (fitvar, 
                        coef=
                        2
                        )

                        # Top 10 differentially variable CpGs between old vs. newborns

                        topDV
                    




                        ## 	      SampleVar LogVarRatio DiffLevene         t 	    P.Value
## cg19078576 1.1128910    3.746586  0.8539180  7.006476 0.0000000006234780
## cg11661000 0.5926226    3.881306  0.8413614  6.945711 0.0000000008176807
## cg07065220 1.0111380    4.181802  0.9204407  6.840327 0.0000000013069867
## cg05995465 1.4478673   -5.524284 -1.3035981 -6.708321 0.0000000023462074
## cg18091046 1.1121511    3.564282  1.0983340  6.679920 0.0000000026599570
## cg05491001 0.9276904    3.869760  0.7118591  6.675892 0.0000000027077013
## cg05542681 1.0287320    3.783637  0.9352814  6.635588 0.0000000032347355
## cg02726803 0.3175570    4.063650  0.6418968  6.607508 0.0000000036608219
## cg08362283 1.0028907    4.783899  0.6970960  6.564472 0.0000000044240941
## cg18160402 0.5624192    3.716228  0.5907985  6.520508 0.0000000053665347
## 	       Adj.P.Value
## cg19078576 0.0001754857
## cg11661000 0.0001754857
## cg07065220 0.0001869984
## cg05995465 0.0001937035
## cg18091046 0.0001937035
## cg05491001 0.0001937035
## cg05542681 0.0001964159
## cg02726803 0.0001964159
## cg08362283 0.0002109939
## cg18160402 0.0002303467
                    


**Figure 14.**
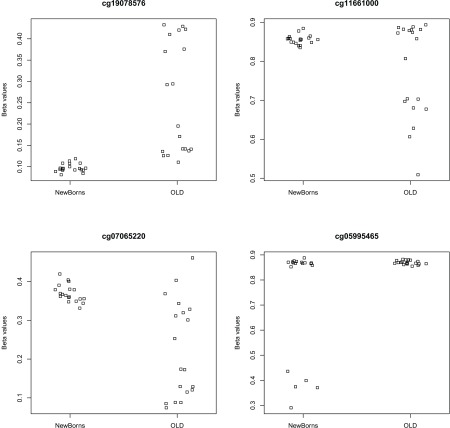
As for DMPs, it is useful to plot the top few differentially variable CpGs to check that the results make sense.



Similarly to the differential methylation analysis, is it useful to plot sample-wise beta values for the differentially variable CpGs to ensure the significant results are not driven by artifacts or outliers ( Figure 14).

                        # get beta values for ageing data

                        age.bVals <- 
                        getBeta
                        (age.mSetSqFlt)
                    




                        par
                        (
                        mfrow=c
                        (
                        2,
                        2
                        ))

                        sapply
                        (
                        rownames
                        (topDV)[
                        1:
                        4
                        ], function(cpg){
  
                        plotCpg
                        (age.bVals, 
                        cpg=
                        cpg, 
                        pheno=
                        age.targets$Sample_Group,
	   
                        ylab = 
                        "Beta values"
                        )
})
                    


An example of testing for differential variability when the design matrix does not have an intercept term is detailed in the *missMethyl* vignette.

### Cell type composition

As methylation is cell type specific and methylation arrays provide CpG methylation values for a population of cells, biological findings from samples that are comprised of a mixture of cell types, such as blood, can be confounded with cell type composition ( Jaffe & Irizarry, 2014). The *minfi* function estimateCellCounts facilitates the estimation of the level of confounding between phenotype and cell type composition in a set of samples. The function uses a modified version of the method published by Houseman *et al.* (2012) and the package FlowSorted.Blood.450k, which contains 450k methylation data from sorted blood cells, to estimate the cell type composition of blood samples.



                        # load sorted blood cell data package

                        library
                        (FlowSorted.Blood.450k)

                        # ensure that the "Slide" column of the rgSet pheno data is numeric
# to avoid "estimateCellCounts" error

                        pData
                        (age.rgSet)$Slide <- 
                        as.numeric
                        (
                        pData
                        (age.rgSet)$Slide)

                        # estimate cell counts

                        cellCounts <- 
                        estimateCellCounts
                        (age.rgSet)
                    




                        
                            *## [estimateCellCounts] Combining user data with reference (flow sorted) data.*

## [estimateCellCounts] Processing user and reference data together.

## [preprocessQuantile] Mapping to genome.

## [preprocessQuantile] Fixing outliers.

## [preprocessQuantile] Quantile normalizing.

## [estimateCellCounts] Picking probes for composition estimation.

## [estimateCellCounts] Estimating composition.
                    




                        # plot cell type proportions by age

                        par
                        (
                        mfrow=c
                        (
                        1
                        ,
                        1
                        ))

                        a = cellCounts[age.targets$Sample_Group == 
                        "NewBorns"
                        ,]

                        b = cellCounts[age.targets$Sample_Group == 
                        "OLD"
                        ,]

                        boxplot
                        (a, 
                        at=
                        0
                        :
                        5
                        *
                        3 
                        + 
                        1
                        , 
                        xlim=c
                        (
                        0
                        , 
                        18
                        ), 
                        ylim=range
                        (a, b), 
                        xaxt=
                        "n"
                        ,
         
                        col=
                        age.pal[
                        1
                        ], 
                        main=
                        ""
                        , 
                        ylab=
                        "Cell type proportion"
                        )

                        boxplot
                        (
                        b
                        , 
                        at=
                        0
                        :
                        5
                        *
                        3 
                        + 
                        2
                        , 
                        xaxt=
                        "n"
                        , 
                        add=
                        TRUE
                        , 
                        col=
                        age.pal
                        [
                        2
                        ])

                        axis
                        (
                        1
                        , 
                        at=
                        0
                        :
                        5
                        *
                        3 
                        + 
                        1.5
                        , 
                        labels=colnames
                        (a), 
                        tick=
                        TRUE
                        )

                        legend
                        (
                        "topleft"
                        , 
                        legend=c
                        (
                        "NewBorns"
                        ,
                        "OLD"
                        ), 
                        fill=
                        age.pal)
                    


**Figure 15.**
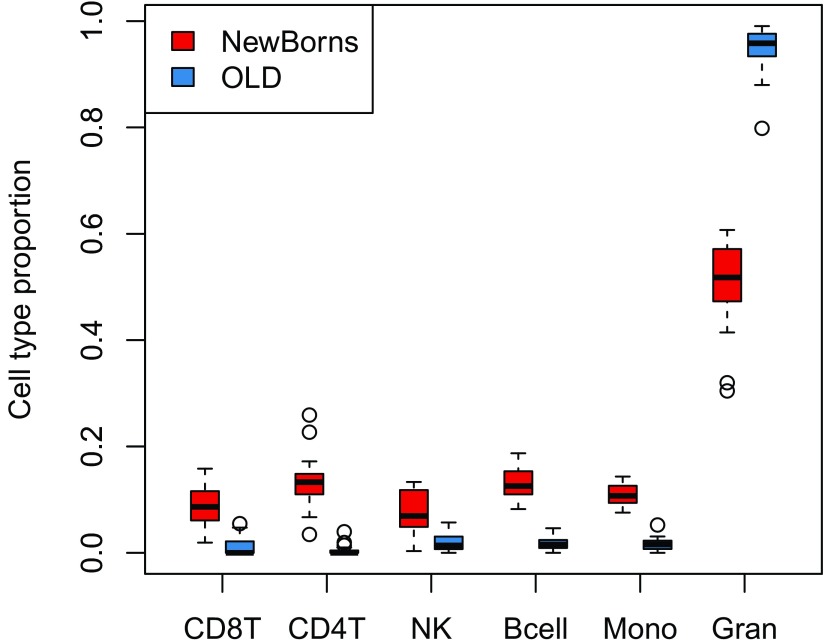
If samples come from a population of mixed cells e.g. blood, it is advisable to check for potential confounding between differences in cell type proportions and the factor of interest.

As reported by Jaffe & Irizarry (2014), the preceding plot demonstrates that differences in blood cell type proportions are strongly confounded with age in this dataset ( Figure 15). Performing cell composition estimation can alert you to potential issues with confounding when analysing a mixed cell type dataset. Based on the results, some type of adjustment for cell type composition may be considered, although a naive cell type adjustment is not recommended. Jaffe & Irizarry (2014) outline several strategies for dealing with cell type composition issues.

## Discussion

Here we present a commonly used workflow for methylation array analysis based on a series of Bio-conductor packages. While we have not included all the possible functions or analysis options that are available for detecting differential methylation, we have demonstrated a common and well used workflow that we regularly use in our own analysis. Specifically, we have not demonstrated more complex types of analyses such as removing unwanted variation in a differential methylation study ( Leek *et al.*, 2012; Maksimovic *et al.*, 2015; Teschendorff *et al.*, 2011), block finding ( Aryee *et al.*, 2014; Hansen *et al.*, 2011) or A/B compartment prediction ( Fortin & Hansen, 2015). Our differential methylation workflow presented here demonstrates how to read in data, perform quality control and filtering, normalisation and differential methylation testing. In addition we demonstrate analysis for differential variability, gene set testing and estimating cell type composition. One important aspect of exploring results of an analysis is visualisation and we also provide an example of generating region-level views of the data.

## Software versions

The R markdown file and/or R script for this version workflow can be downloaded from: https://github.com/Oshlack/MethylationAnalysisWorkflow/tree/version3/scripts. A *live* version of this workflow that is regularly built using updated Bioconductor packages can be accessed on the Bioconductor website: https://www.bioconductor.org/help/workflows/methylationArrayAnalysis/. The version of the workflow presented here uses the following packages available from Bioconductor (release 3.4):



                    sessionInfo
                    ()


                    ## R version 3.3.1 (2016-06-21)
## Platform: x86_64-pc-linux-gnu (64-bit)
## Running under: CentOS release 6.7 (Final)
##
## locale:
##  [1] LC_CTYPE=en_US.UTF-8       LC_NUMERIC=C
##  [3] LC_TIME=en_US.UTF-8        LC_COLLATE=en_US.UTF-8
##  [5] LC_MONETARY=en_US.UTF-8    LC_MESSAGES=en_US.UTF-8
##  [7] LC_PAPER=en_US.UTF-8       LC_NAME=C
##  [9] LC_ADDRESS=C               LC_TELEPHONE=C
## [11] LC_MEASUREMENT=en_US.UTF-8 LC_IDENTIFICATION=C
##
## attached base packages:
##  [1] splines   grid      stats4    parallel  stats     graphics  grDevices
##  [8] utils     datasets  methods   base
##
## other attached packages:
##  [1] stringr_1.2.0
##  [2] DMRcate_1.10.8
##  [3] DMRcatedata_1.10.1
##  [4] DSS_2.14.0
##  [5] bsseq_1.10.0
##  [6] Gviz_1.18.2
##  [7] minfiData_0.20.0
##  [8] matrixStats_0.51.0
##  [9] missMethyl_1.8.0
## [10] RColorBrewer_1.1-2
## [11] IlluminaHumanMethylation450kmanifest_0.4.0
## [12] IlluminaHumanMethylation450kanno.ilmn12.hg19_0.6.0
## [13] minfi_1.20.2
## [14] bumphunter_1.14.0
## [15] locfit_1.5-9.1
## [16] iterators_1.0.8
## [17] foreach_1.4.3
## [18] Biostrings_2.42.1
## [19] XVector_0.14.1
## [20] SummarizedExperiment_1.4.0
## [21] GenomicRanges_1.26.4
## [22] GenomeInfoDb_1.10.3
## [23] IRanges_2.8.2
## [24] S4Vectors_0.12.2
## [25] Biobase_2.34.0
## [26] BiocGenerics_0.20.0
## [27] limma_3.30.13
##
## loaded via a namespace (and not attached):
##   [1] colorspace_1.3-2
##   [2] siggenes_1.48.0
##   [3] mclust_5.2.3
##   [4] rprojroot_1.2
##   [5] biovizBase_1.22.0
##   [6] htmlTable_1.9
##   [7] base64enc_0.1-3
##   [8] dichromat_2.0-0
##   [9] base64_2.0
##  [10] interactiveDisplayBase_1.12.0
##  [11] AnnotationDbi_1.36.2
##  [12] IlluminaHumanMethylationEPICanno.ilm10b2.hg19_0.6.0
##  [13] codetools_0.2-15
##  [14] R.methodsS3_1.7.1
##  [15] methylumi_2.20.0
##  [16] knitr_1.15.1
##  [17] Formula_1.2-1
##  [18] Rsamtools_1.26.1
##  [19] annotate_1.52.1
##  [20] cluster_2.0.5
##  [21] GO.db_3.4.0
##  [22] R.oo_1.21.0
##  [23] shiny_1.0.0
##  [24] httr_1.2.1
##  [25] backports_1.0.5
##  [26] assertthat_0.1
##  [27] Matrix_1.2-8
##  [28] lazyeval_0.2.0
##  [29] acepack_1.4.1
##  [30] htmltools_0.3.5
##  [31] tools_3.3.1
##  [32] gtable_0.2.0
##  [33] doRNG_1.6
##  [34] Rcpp_0.12.9
##  [35] multtest_2.30.0
##  [36] preprocessCore_1.36.0
##  [37] nlme_3.1-131
##  [38] rtracklayer_1.34.2
##  [39] mime_0.5
##  [40] ensembldb_1.6.2
##  [41] rngtools_1.2.4
##  [42] gtools_3.5.0
##  [43] statmod_1.4.29
##  [44] XML_3.98-1.5
##  [45] beanplot_1.2
##  [46] org.Hs.eg.db_3.4.0
##  [47] AnnotationHub_2.6.5
##  [48] zlibbioc_1.20.0
##  [49] MASS_7.3-45
##  [50] scales_0.4.1
##  [51] BSgenome_1.42.0
##  [52] VariantAnnotation_1.20.3
##  [53] BiocInstaller_1.24.0
##  [54] GEOquery_2.40.0
##  [55] yaml_2.1.14
##  [56] memoise_1.0.0
##  [57] gridExtra_2.2.1
##  [58] ggplot2_2.2.1
##  [59] pkgmaker_0.22
##  [60] biomaRt_2.30.0
##  [61] rpart_4.1-10
##  [62] reshape_0.8.6
##  [63] latticeExtra_0.6-28
##  [64] stringi_1.1.2
##  [65] RSQLite_1.1-2
##  [66] highr_0.6
##  [67] genefilter_1.56.0
##  [68] permute_0.9-4
##  [69] checkmate_1.8.2
##  [70] GenomicFeatures_1.26.3
##  [71] BiocParallel_1.8.1
##  [72] bitops_1.0-6
##  [73] nor1mix_1.2-2
##  [74] evaluate_0.10
##  [75] lattice_0.20-34
##  [76] ruv_0.9.6
##  [77] GenomicAlignments_1.10.1
##  [78] htmlwidgets_0.8
##  [79] plyr_1.8.4
##  [80] magrittr_1.5
##  [81] R6_2.2.0
##  [82] Hmisc_4.0-2
##  [83] DBI_0.6
##  [84] foreign_0.8-67
##  [85] survival_2.40-1
##  [86] RCurl_1.95-4.8
##  [87] nnet_7.3-12
##  [88] tibble_1.2
##  [89] rmarkdown_1.3
##  [90] data.table_1.10.4
##  [91] digest_0.6.12
##  [92] xtable_1.8-2
##  [93] httpuv_1.3.3
##  [94] illuminaio_0.16.0
##  [95] R.utils_2.5.0
##  [96] openssl_0.9.6
##  [97] munsell_0.4.3
##  [98] registry_0.3
##  [99] BiasedUrn_1.07
## [100] quadprog_1.5-5
				

