# Supplementary material for: A cross-package Bioconductor workflow for analysing methylation array data
Source: F1000Res. 2017 Apr 5;5:1281. Originally published 2016 Jun 8. [Version 3] doi: 10.12688/f1000research.8839.3 (PMC4916993; doi:10.12688/f1000research.8839.3)
Supplement: Peer review history

# Peer review: Referee response for version 2

**DOI:** 10.5256/f1000research.9951.r17311

**Published:** 2016-10-31

**Authors:** Timothy J. Peters

**Affiliations:** 1 Garvan Institute of Medical Research, Darlinghurst, NSW, Australia

I am happy with the amendments made, and approve of the publication.

I have read this submission. I believe that I have an appropriate level of expertise to confirm that it is of an acceptable scientific standard.

# Peer review: Referee response for version 2

**DOI:** 10.5256/f1000research.9951.r15416

**Published:** 2016-08-03

**Authors:** Peter Hickey

**Affiliations:** 1 Johns Hopkins Bloomberg School of Public Health, Baltimore, MD, USA

Thank you for making the data easily available and re-running the workflow with the latest release version of Bioconductor, Jovanna. My minor comments on the initial submission have also been addressed to my satisfaction. Therefore, I happily approve the revised manuscript. I believe it will be a helpful resource to the Bioconductor community and those wishing to learn best practises for analysing DNA methylation array data.

 Minor comments I have on the revised manuscript:

- I got slightly different numbers and output when running the workflow (see below). I believe this is due to a bug being fixed in in v1.18.4 (see https://github.com/Bioconductor-mirror/minfi/commit/c16b28e2f4c7127696cbea807f668d0a10c54b37); I tested using minfi v1.18.6. These differences were first observable when running This affects all subsequent output such as which probes are identified as DMPs. Unfortunately, such changes in output are par for the course in bioinformatics software. These differences are outside the author's control, and I do not believe these affect the quality and usefulness of the workflow. I'm not sure anything can be done except to point out that such differences may exist due to different software versions.

> head(mVals[,1:5])                      

                        naive.1     rTreg.2  act_naive.3    naive.4  act_naive.5

 cg13869341  2.205808  2.205808    2.167697  2.173122    2.106660

 cg24669183  2.169414  2.235964    2.280734  1.632309    2.184435

 cg15560884  1.761176  1.577578    1.597503  1.777486    1.764999

 cg01014490 -2.918305 -2.979692   -4.042347 -3.448734   -3.189924

 cg17505339  3.082191  3.924931    4.163206  3.255373    3.654134

 cg11954957  1.546401  1.912204    1.727910  2.441267    1.618331

  

- There is a typo (extra whitespace) in the BiocViews URL
- I was unable to get the Gviz plot to correctly render. Unsure what's going on here as the Gviz version on my machine matches that in the workflow (v1.16.1)

> plotTracks(tracks, from=minbase, to=maxbase, showTitle=TRUE, add53=TRUE,

 +            add35=TRUE, grid=TRUE, lty.grid=3, sizes=sizes, length(tracks))

 Error in valid.viewport(x, y, width, height, just, gp, clip, xscale, yscale,  :

   invalid 'yscale' in viewport

 In addition: Warning messages:

 1: In min(x) : no non-missing arguments to min; returning Inf

 2: In max(x) : no non-missing arguments to max; returning -Inf

I have read this submission. I believe that I have an appropriate level of expertise to confirm that it is of an acceptable scientific standard.

# Peer review: Referee response for version 1

**DOI:** 10.5256/f1000research.9514.r14247

**Published:** 2016-06-22

**Authors:** Davide Risso

**Affiliations:** 1 Division of Biostatistics, School of Public Health, University of California, Berkeley, Berkeley, CA, USA

As someone who has experience with R/Bioconductor and with genomics data, but not direct experience analyzing methylation array data, I found the workflow very useful and I would suggest it to anyone wanting to start analyzing this type of data.

 I do agree with the other reviewers that the value of the workflow will be greatly increased if the dataset used was available as an R object. The authors should consider submitting an experiment data package to Bioconductor to accompany the workflow. Alternatively, they could provide the dataset as a supplementary file.

 As for the analysis itself, I only have one major question. Note that I do not have direct experience analyzing methylation array, so this is a genuine question rather than a criticism.

 In gene expression analysis, we tend to perform filtering prior to normalization, while the authors here first normalize the data by quantile normalization and then filter out probes that are low quality and/or affected by SNPs. Wouldn't it be safer to perform filtering before normalization? I understand that given the few probes affected, the order has likely very little effect in this dataset. But I naively imagine that if there are many problematic probes and, say, the quality of the samples is confounded with the biology, there could be issues in using low quality probes for normalization.

 Other minor points:

- I agree that the code should be re-run with the latest release of R and Bioconductor.
- In the definition of \beta, \alpha should be defined, and its default value in getBeta() should be specified.
- Spelling: most of the article uses British English spelling, but the word "normalization" is sometimes (but not always) spelled in American English.
- A sentence describing what is the procedure implemented in preprocessQuantile() is needed for people not familiar with normalization.
- I agree that it would be useful to provide a brief description of what is a contrasts matrix as this section could be confusing for people unfamiliar with statistical models.
- For the same reason, the authors should add a brief explanation of the problem of multiple testing and what is the false discovery rate. Or at least provide references to the appropriate literature.

I have read this submission. I believe that I have an appropriate level of expertise to confirm that it is of an acceptable scientific standard.

# Response

**Published:** 2016-07-14

**Authors:** Jovana Maksimovic

**Affiliations:** Murdoch Childrens Research Institute, Australia

Thanks for your review, Davide. 

 While we agree that normalisation post-filtering makes sense, there are some practical aspects with the data objects that minfi uses which makes this difficult. Many (but not all) normalisation procedures in minfi accept an rgSet object, which can be thought of as a raw data object, which cannot easily be subset by CpG site. These normalisation procedures then output a different type of data object, such as MethylSet or GenomicRatioSet, which are much easier to work with in terms of filtering out problematic CpG sites. Due to the sheer number of CpG sites observed per sample (>450,000) we believe it shouldn’t make too much difference for most datasets, especially if very poor quality samples are excluded prior to normalisation, although it is possible that there are exceptions to this.

 Response to minor points:

- We have spent some time modifying the workflow to run with the latest R and Bioconductor.
- We have added additional details regarding beta values, M-values and the offset in the paper. 
- We have changed "normalization" to "normalisation" throughout the text.
- A sentence has been added about preprocessQuantile in the normalisation section. 
- We have included some additional explanation of contrast matrices. 
- An additional paragraph was added explaining about the issues of multiple testing in very high dimensional data.

# Peer review: Referee response for version 1

**DOI:** 10.5256/f1000research.9514.r14248

**Published:** 2016-06-21

**Authors:** Michael I. Love

**Affiliations:** 1 Department of Biostatistics, Harvard TH Chan School of Public Health, Boston, MA, USA

I am not an expert in analysis of methylation data, and have not used the methylation packages mentioned in this workflow, so I reviewed the workflow as an uninitiated reader might approach it.

 Major comments:

 I found the workflow to be easy to follow and informative. The authors have done a good job summarizing a large and complex topic into an reasonable size for a workflow article, while still mentioning the various alternatives that are possible at each step. I appreciated the focus on EDA and checking the quality of results by eye, for example the M-values for the most significant tests and the MDS plots colored by different variables.

 I did not try to run the code, and I agree with the other two reviewers that the code and datasets should be made available and linked to from this workflow.

 Minor comments:

 The first time “moderated t-statistics” is mentioned, it would benefit to have a citation so that a reader who hasn’t encountered this method before can read the reference, e.g. Smyth 2004.

 The first or second time IDAT files are mentioned, a small description of these would be useful, a little more than just that these are the raw files. Which platforms produce IDAT files? Are they compressed files? About how large are they?

 Figure 2: It wasn’t obvious at first that the plot on the right is the same as the left but zoomed in.

 When discussing the choice of normalization depending on whether or not there are global changes across samples due to underlying biology, the authors might consider referencing the *quantro* article and Bioconductor package by Stephanie Hicks for determining whether there are global changes in genomic datasets across samples, and therefore whether quantile normalization is appropriate. Hicks has an example of whether or not to use quantile normalization for methylation data in the article.

- https://www.bioconductor.org/packages/quantro

Principal components is misspelled in the text: “principle components”

 In the paragraph above the call to *makeContrasts*, it would be good to state in the text in one sentence what it is this function does, for the benefit of someone who has never performed linear modeling before. Likewise, to explicitly state that coef=1 is referencing the first column of the contrast matrix. It should be stated what is the B-statistic which orders the topTable.

 The authors should explain a bit more what is being shown in Figure 10 in the caption.

 In the text and code the authors have written DNAseI, but I believe the more common capitalization is DNaseI.

 The authors might consider commenting on the top GO categories and the associated FDR values. How far down the list should one look? Can the authors advise the reader how GO results should be reported? Is it fair to pick out the most relevant categories from this list and only report them?

 It wasn’t clear to me the difference between the *gometh* and *gsameth* approaches.

 It would be good to provide references to literature for “it has been hypothesised that highly variable CpGs in cancer are important for tumour progression”.

I have read this submission. I believe that I have an appropriate level of expertise to confirm that it is of an acceptable scientific standard.

## References

[1] SmythGK: Linear models and empirical bayes methods for assessing differential expression in microarray experiments. *Stat Appl Genet Mol Biol*.2004;3: 10.2202/1544-6115.1027 Article3 10.2202/1544-6115.1027 16646809

[2] HicksSCIrizarryRA: quantro: a data-driven approach to guide the choice of an appropriate normalization method. *Genome Biol*.2015;16: 10.1186/s13059-015-0679-0 117 10.1186/s13059-015-0679-0 26040460PMC4495646

# Response

**Published:** 2016-07-14

**Authors:** Jovana Maksimovic

**Affiliations:** Murdoch Childrens Research Institute, Australia

Thank you for reviewing our paper, Michael.

 In response to your comments/suggestions we have made the following changes:

- The Smyth 2004 citations have been added the first time “moderated t-statistics” is mentioned
- A description of IDAT files has been added to the text along with a reference to a Bioconductor package that is specifically for reading IDAT files.
- We have added to the legend for Figure 2 to clarify that the plot on the right is the same as the left but zoomed in.
- There is now a reference to Hicks and quantro in included in the Normalisation section.
- Spelling mistakes and typos have been fixed.
- Function of *makeContrasts* is described: See response to Davide Risso.
- We now explicitly state that coef=1 is referencing the first column of the contrast matrix.
- Included explanation for B-statistic and citation.
- More detail about the plot has been added to the figure caption for Figure 10.
- Changed DNAseI to DNasel
- Typically we would consider GO categories that have associated FDRs less than 5% as significant. Some discussion of these points has been added to the gene set testing section.
- The gometh function specifically tests only GO and KEGG pathways, whereas the gsameth is a more general function that requires the user to supply their own gene sets for testing.
- We have changed the sentence “it has been hypothesised that highly variable CpGs in cancer are important for tumour progression” to “it has been hypothesised that highly variable CpGs in cancer may contribute to tumour heterogeneity” and included the following reference:
  Hansen KD, Timp W, Bravo HC, Sabunciyan S, Langmead B, McDonald OG, Wen B, Wu H, Liu Y, Diep D, Briem E, Zhang K, Irizarry RA, Feinberg AP: Increased methylation variation in epigenetic domains across cancer types. Nat Genet. 2011, 43: 768-775

# Peer review: Referee response for version 1

**DOI:** 10.5256/f1000research.9514.r14425

**Published:** 2016-06-17

**Authors:** Peter Hickey

**Affiliations:** 1 Johns Hopkins Bloomberg School of Public Health, Baltimore, MD, USA

This paper is a well-written workflow for analysing DNA methylation microarrays using Bioconductor packages. A challenge in writing these workflows is to produce something that is opinionated enough to be useful and balanced enough to be fair to packages developed by other people; I believe the authors have struck the right balance.

 However, my overall assessment is "Approved With Reservations" because the data used in the workflow is not easily available and therefore the workflow cannot be tested out by the interested reader.

 I spent some time trying to compile the raw data from GEO, but to me this feels a bit too much to expect of the reader, especially when it is likely that the interested reader is a beginner or intermediate user of bioinformatics software. I strongly believe the workflow should either include code to curate/construct/download the necessary files such as SampleSheet.csv and the IDAT files or include a link to prepared example data that can be used right from the 'Loading the data' section of the workflow. For example, http://f1000research.com/articles/4-1070/v1 uses data from the *airway* Bioconductor package that can easily be installed by the reader to follow along with the workflow.

 My other main suggestion would be to re-run the code using the recently published Bioconductor version 3.3. I expect this might require some minor changes to the code, e.g., the minfi::read.450k* functions have been deprecated in favour of minfi::read.metharray* functions.

 I have some additional minor comments and suggestions that I will include once I'm able to run through and review the workflow from beginning to end.

I have read this submission. I believe that I have an appropriate level of expertise to confirm that it is of an acceptable scientific standard, however I have significant reservations, as outlined above.

# Response

**Published:** 2016-07-06

**Authors:** Peter Hickey

**Affiliations:** Johns Hopkins Bloomberg School of Public Health, USA

- p2: beta = M / (M + U + alpha), the alpha parameter should be explained. Also, both the definition of beta and Mvalue differ slightly from that given in the cited Du, P. et al. Comparison of Beta-value and M-value methods for quantifying methylation levels by microarray analysis. BMC Bioinformatics 11, 587 (2010).
- p3: Perhaps worth mentioning that a complete list of packages for analysing DNA methylation data can be accessed using BiocViews (https://www.bioconductor.org/packages/release/bioc/html/biocViews.html and https://www.bioconductor.org/packages/release/BiocViews.html#___DNAMethylation)
- p4: "...loading all the package libraries..." should be "...loading all the packages..."
- p4: Perhaps worth commenting on which of the loaded packages are methylation-focused and/or purpose of other packages, e.g., stringr, Gviz.
- p4: This is *super* pedantic (sorry!): strictly speaking the IlluminaHumanMethylation450kmanifest package provides the Illumina manifest for the 450k array, which can then be accessed by using `minfi::getAnnotation()`
- Figure 2: Not immediately obvious that righthand plot is zoomed in version of lefthand plot. The caption could better explain this.
- p10: The code produces a warning. Would be helpful to the reader to comment on whether this is cause for concern in this case.
- Figure 9: Wondering whether helpful to have each panel with y-axis = [0, 1]
- p25: `islandData` apparently contains 0 ranges. This looks like a bug in the code.
- p28: "For gene ontology testing (default), the user can specific collection = "GO" for KEGG testing collection = "KEGG""; this sentence seems incomplete or is perhaps missing a word
- p29 and p30: The code produces a warning. Would be helpful to the reader to comment on whether this is cause for concern in this case.
- The workflow uses multiple packages and it's not always clear where each function comes from. This could be clarified e.g., by namespacing functions such as `limma::plotMDS()` instead of `plotMDS()`

# Response

**Published:** 2016-07-14

**Authors:** Jovana Maksimovic

**Affiliations:** Murdoch Childrens Research Institute, Australia

Thanks for taking the time to review our workflow, Peter.

 In response to your suggestion we have made the data available and rerun the workflow using the latest R and Bioconductor.

 In response to your other comments: 

- p2: beta = M / (M + U + alpha), the alpha parameter should be explained. Also, both the definition of beta and Mvalue differ slightly from that given in the cited Du, P. et al. Comparison of Beta-value and M-value methods for quantifying methylation levels by microarray analysis. BMC Bioinformatics 11, 587 (2010).
*This has been addressed. See response to Davide Risso.*
- p3: Perhaps worth mentioning that a complete list of packages for analysing DNA methylation data can be accessed using BiocViews (https://www.bioconductor.org/packages/release/bioc/html/biocViews.html and https://www.bioconductor.org/packages/release/BiocViews.html#___DNAMethylation)
*This has been added to the paper.*
- p4: "...loading all the package libraries..." should be "...loading all the packages..."
*The text has been modified accordingly.*
- p4: Perhaps worth commenting on which of the loaded packages are methylation-focused and/or purpose of other packages, e.g., stringr, Gviz.
*The text has been modified accordingly.*
- p4: This is *super* pedantic (sorry!): strictly speaking the IlluminaHumanMethylation450kmanifest package provides the Illumina manifest for the 450k array, which can then be accessed by using `minfi::getAnnotation()`
*The text has been modified accordingly.*
- Figure 2: Not immediately obvious that righthand plot is zoomed in version of lefthand plot. The caption could better explain this.
*This has been clarified in the figure caption.*
- p10: The code produces a warning. Would be helpful to the reader to comment on whether this is cause for concern in this case.
*A sentence has been included that explains the reason for the waring.*
- Figure 9: Wondering whether helpful to have each panel with y-axis = [0, 1]
*As we are trying to highlight the differences between the groups tested for **individual** CpGs and not comparing between CpGs, we feel that the axes are appropriate for the purposes of "sanity checking" the results of the statistical analysis.*
- p25: `islandData` apparently contains 0 ranges. This looks like a bug in the code.
*This was due to the fact that there were not any CpG islands present in the region being plotted; we have selected another region to plot that does have a CpG island so that islandData is no longer empty.*
- p28: "For gene ontology testing (default), the user can specific collection = "GO" for KEGG testing collection = "KEGG""; this sentence seems incomplete or is perhaps missing a word
*This sentence has been modified.*
- p29 and p30: The code produces a warning. Would be helpful to the reader to comment on whether this is cause for concern in this case.
*Added a sentence to the text to explain the warning.*
- The workflow uses multiple packages and it's not always clear where each function comes from. This could be clarified e.g., by namespacing functions such as `limma::plotMDS()` instead of `plotMDS()`
*We don’t feel it is a particularly useful exercise to change every function to include the package name. Searching the help for any of the functions will inform users which package the function comes from. For example ?plotMDS.*

# Peer review: Referee response for version 1

**DOI:** 10.5256/f1000research.9514.r14245

**Published:** 2016-06-15

**Authors:** Timothy J. Peters

**Affiliations:** 1 Garvan Institute of Medical Research, Darlinghurst, NSW, Australia

This paper describes a workflow for processing, filtering and analysis of Illumina Infinium methylation array data. It showcases a reproducible pipeline integrating a suite of tools from Bioconductor for multi-faceted genomic insights. While none of the tools individually are novel, their integration into a sensible, reproducible pipeline is. I am recommending this manuscript for indexationfor 3 main reasons:

- The tools contained therein and their application are in line with, or near, best practice. The workflow itself contains all the major steps that this reviewer usually uses for their methylation array processing.
- An integrated workflow such as this will be valuable for novice and intermediate bioinformaticians who are tasked with processing methylation data. The number of caveats and sanity checks needed for appropriate biological interpretation is not trivial, and this workflow does a satisfactory job of outlining them.
- The reproducible nature of this manuscript is a strength; it is very "coalface bioinformatics". Many published methods have very poor or buggy implementations and no effort is made to contextualise them in a given pipeline. Publication may set a precedent for other authors to give worked examples and context, which in this reviewer's opinion accelerates the path to best practice.

Minor amendments needed:

I could not find any public links to the data files imported into this workflow. These ought to be provided.
a) The mathematical definition of *β* is given as *β = M/(M +U + α)*. While I realise *α* is a fudge factor for offset purposes this is not clear to the lay reader and needs to be made so.
  b) Why are there no offsets for *M* or *U *in the calculation of *M *values, especially since there is one for the calculation of *β*? On (admittedly) rare occasions *M* or *U *will be exactly zero and hence offsets need to be put in both the numerator and denominator of the ratio to be log-transformed, else a non-number will result.
A justification for the preference of *M* values over *β *values for use in the MDS plots is needed, especially since the statement is made that "Beta values are generally preferable ... for graphical presentation". This reviewer's experience is that *β *is much more common for use in PCA/MDS, and is certainly the standard for other methylation platforms e.g. bisulfite sequencing data.
Legends are needed for density plots in Figs. 3 and 8. I appreciate *minfi* annoyingly puts the default legend in the top right, obscuring the hypermethylated mode, but a custom call to legend() ought to fix this.
Appropriate Y-axis labels are needed for Figs. 9 and 14.

I have read this submission. I believe that I have an appropriate level of expertise to confirm that it is of an acceptable scientific standard.

# Response

**Published:** 2016-07-14

**Authors:** Jovana Maksimovic

**Affiliations:** Murdoch Childrens Research Institute, Australia

Thanks Tim for taking the time to review our paper.

 In response to your comments/suggestions:

- I could not find any public links to the data files imported into this workflow. These ought to be provided.
*In addition to the references, we have now included links to GEO for the data used and have also made a bundle of all the data available on Figshare which can now be used directly from within R to download the data and complete the workflow. *
- a) The mathematical definition of *β* is given as *β = M/(M +U + α)*. While I realise *α* is a fudge factor for offset purposes this is not clear to the lay reader and needs to be made so.
  b) Why are there no offsets for *M* or *U *in the calculation of *M *values, especially since there is one for the calculation of *β*? On (admittedly) rare occasions *M* or *U *will be exactly zero and hence offsets need to be put in both the numerator and denominator of the ratio to be log-transformed, else a non-number will result.
*This has been clarified in the text. See also response to Davide Risso.*
- A justification for the preference of *M* values over *β *values for use in the MDS plots is needed, especially since the statement is made that "Beta values are generally preferable ... for graphical presentation". This reviewer's experience is that *β *is much more common for use in PCA/MDS, and is certainly the standard for other methylation platforms e.g. bisulfite sequencing data.
*We disagree that beta values should be used in principal components analysis. While plotMDS does produce a graphic, the function is performing a statistical analysis (i.e. principal components analysis), which is based on normal distribution theory. The same reasons for not performing differential methylation analysis on the beta values apply in this case (i.e. heteroscedasticity of the beta values).  *
- Legends are needed for density plots in Figs. 3 and 8. I appreciate *minfi* annoyingly puts the default legend in the top right, obscuring the hypermethylated mode, but a custom call to legend() ought to fix this.
*These legends have been added as suggested.*
- Appropriate Y-axis labels are needed for Figs. 9 and 14.
*The Y-axis labels have been added.*
